# HIV-1 Nef Impairs Key Functional Activities in Human Macrophages through CD36 Downregulation

**DOI:** 10.1371/journal.pone.0093699

**Published:** 2014-04-04

**Authors:** Eleonora Olivetta, Valentina Tirelli, Chiara Chiozzini, Beatrice Scazzocchio, Ignazio Romano, Claudia Arenaccio, Massimo Sanchez

**Affiliations:** 1 National AIDS Centre, Istituto Superiore di Sanità, Rome, Italy; 2 Department of Cell Biology and Neurosciences, Istituto Superiore di Sanità, Rome, Italy; 3 Department of Veterinary, Public Health and Food Safety, Istituto Superiore di Sanità, Rome, Italy; 4 Department of Science, University Roma TRE, Rome, Italy; Ghent University, Belgium

## Abstract

Monocytes and macrophages utilize the class A and B scavenger receptors to recognize and perform phagocytosis of invading microbes before a pathogen-specific immune response is generated. HIV-1 Nef protein affects the innate immune system impairing oxidative burst response and phagocytic capacity of macrophages. Our data show that exogenous recombinant myristoylated Nef protein induces a marked CD36 downregulation in monocytes from Peripheral Blood Mononuclear Cells, in Monocyte-Derived Macrophages (MDMs) differentiated by cytokines and in MDMs contained in a mixed culture obtained expanding PBMCs under Human Erythroid Massive Amplification condition. Under the latter culture condition we identify three main populations after 6 days of expansion: lymphocytes (37.8±14.7%), erythroblasts (46.7±6.1%) and MDMs (15.7±7.5%). The Nef addition to the cell culture significantly downregulates CD36 expression in MDMs, but not in erythroid cells. Furthermore, CD36 inhibition is highly specific since it does not modify the expression levels of other MDM markers such as CD14, CD11c, CD86, CD68, CD206, Toll-like Receptor 2 and Toll-like Receptor 4. Similar results were obtained in MDMs infected with VSV-G pseudotyped HIV-1-expressing Nef. The reduced CD36 membrane expression is associated with decrease of correspondent CD36 mRNA transcript. Furthermore, Nef-induced CD36 downregulation is linked to both impaired scavenger activity with reduced capability to take up oxidized lipoproteins and to significant decreased phagocytosis of fluorescent beads and GFP-expressing *Salmonella tiphymurium*. In addition we observed that Nef induces TNF-α release in MDMs. Although these data suggest a possible involvement of TNF-α in mediating Nef activity, our results exclude a possible relationship between Nef-induced TNF-α release and Nef-mediated CD36 downregulation. The present work shows that HIV-1 Nef protein may have a role in the strategies elaborated by HIV-1 to alter pathogen disease outcomes, by modulating CD36 expression in macrophages, favoring the onset of opportunistic infections in HIV-1 infected people.

## Introduction

Human phagocytic cells (i.e. monocytes, macrophages and neutrophils) represent the first line of host defences against pathogens, and play a crucial role in removing apoptotic and necrotic cells, and in killing tumor cells [1]. Internalization and subsequent destruction of pathogens are key processes of innate immune response promoting antigen presentation and development of adaptive immunity. In particular, macrophages express activation surface markers and produce proinflammatory cytokines and chemokines to control pathogen spreading by recruiting peripheral lymphocytes and monocytes at the site of inflammation. Monocytes and/or macrophages may also employ Pattern-Recognition Receptors (PRRs) to identify highly conserved microbial structures and to internalize invading microbes before a pathogen-specific immune response has been generated.

The class B scavenger receptor CD36 is an 88-kDa cell surface transmembrane glycoprotein belonging to PRR family that comprises class A and B scavenger receptors. The receptor consists of two short intracellular domains and a large extracellular domain and shows an extensive N-glycosylation; its transcription is mainly controlled by the heterodimer PPARγ/RXR (Peroxisome Proliferator-Activated Receptor γ/Retinoid × Receptor) [2]. CD36 is widely found in different cells and tissues such as macrophages, microglia, microvascular endothelium, cardiac and skeletal muscles, adipocytes and platelets [3]. It is implicated in a wide array of normal and pathologic biological functions, including angiogenesis, atherosclerosis, inflammation, and lipid metabolism [3], [4]. CD36 was first identified as a receptor for recognizing and internalizing specific oxidized phospholipids and lipoproteins, but it also participates in the internalization of apoptotic cells, bacterial and fungal pathogens [5], [6]. As regards bacterial phagocytic function, conflicting results about the specificity of CD36 as a pattern recognition receptor of Gram-positive or Gram-negative bacteria were reported [7], [8]. In this perspective, Baranova et al [9] observed phagocytosis of both Gram-negative and Gram-positive bacteria in hCD36-overexpressing transfected HeLa cells suggesting that no preference exists for their uptake.

Human Immunodeficiency Viruses (HIV-1 and HIV-2) cause Acquired Immunodeficiency Syndrome (AIDS), primarily infecting crucial cells of the immune system such as CD4 T-cells, dendritic and macrophages cells. Before the AntiRetroviral Therapy (ART) era, the role of HIV-1-infected Monocyte-Derived Macrophages (MDMs) in the development of AIDS was unclear. However, it is now evident that the occurrence of macrophage-mediated diseases represents a continuous risk in HIV-1-infected individuals, even in the presence of high counts of CD4^+^ T-cells.

Several HIV-1-associated diseases – i.e. AIDS-Related Lymphoma (ARL), metabolic syndromes (including CardioVascular Diseases, CVDs; atherosclerosis and other lipid disorders), and HIV-Associated Dementia (HAD) – can be considered as all macrophage-mediated disorders in which Nef is an unquestioned key factor [10]. The viral regulatory protein Nef is a 27–34 kDa myristoylated protein produced exclusively by HIV and SIV (Simian Immunodeficiency Virus) and it is considered a virus component that plays a critical role in AIDS pathogenesis in HIV-infected humans [11]. Although Nef does not show catalytic activity, it influences cellular signaling pathways leading to the enhancement of viral replication, immune elusion, and enhanced survival in T-cells and macrophages [12]. At first the functions attributed to Nef were the capacity to down-modulate surface expression of the HIV-1 receptor CD4 and the Major Histocompatibility Complex class I (MHC-I) molecules. Further studies have demonstrated the involvement of Nef protein in dysregulation of HIV-1-infected macrophages functions. In addition to CD4 and MHC-I, other molecules of relevance are modulated by Nef in monocytes/macrophages, included the CCR5, one of the major HIV co-receptors. Nef can also modify signaling pathways in infected as well in non-infected macrophages when captured exogenously as a soluble factor. Other mechanisms based on cell-to-cell transfer are well documented phenomena in macrophage cells as a way to deliver Nef. Indeed, infected macrophages may transfer Nef to B cells, where it would interfere with immunoglobulin class-switch recombination thus contributing to the B-cell dysfunction and humoral defect observed in HIV-1 positive subjects. Furthermore, Nef can protect the infected macrophage from cell death favoring viral production and long-standing persistence specifically inhibiting late maturating stages of autophagosomal pathway. A summary of Nef functions has been recently published by Ghiglione and Turk in a comprehensive review [13] where the Nef biology and its role in HIV pathogenesis are extensively discussed.

HIV-1 infection also compromises the functionality of phagocytic cells favoring the reactivation and development of opportunistic infections during AIDS progression. Nef protein can affect the innate immune system impairing oxidative burst response and phagocytosis in monocytes/macrophages from HIV-1 patients [14]–[16]. Moreover, Nef induces the secretion from primary human monocyte/macrophages of chemotactic factors like the CC-chemokines CCL2 and CCL4 [17], which correlate with the activation of AP-1, NF-κB, STAT1 and STAT3 transcription factors [18]–[22].

In this study we want extend our investigation on the role played by Nef in favoring opportunistic infections during AIDS progression, by clarifying the mechanism of Nef-induced impairment of bacterial pathogen phagocytosis and of other macrophage functions. We provide clear evidence that recombinant Nef (rNef) dramatically reduces the membrane expression of the scavenger receptor CD36 inhibiting oxidized lipoprotein (oxLDL) uptake and impairing *Salmonella tiphymurium* phagocytosis in primary human MDMs.

## Materials and Methods

### Ethic Statement

PBMCs (Peripheral Blood Mononuclear Cells) and LDLs utilized in this study were obtained from buffy coats and pooled fresh plasma of healthy blood donors as anonymously provided by the Immunohematology and Transfusional Center of Policlinico Umberto I, Sapienza University, Rome. All the subjects gave their written informed consent for research purposes according to the Italian law on this matter by the Transfusion Center (Legislative Decree of the Italian Ministry of Health, January 25, 2001 and published in the Official Gazette of April 3, 2001).

### Preparation of PBMCs

PBMCs were isolated by density gradient centrifugation 400 g for 30 min at room temperature over Ficoll-Hypaque (ρ<1.077, Amersham Pharmacia Biotec, Uppsala, Sweden).

### Ex vivo Expansion of PBMCs

Cells were incubated at 37°C in 5% CO_2_ atmosphere and expanded in HEMA (Human Erythroid Massive Amplification) culture, as described by Migliaccio et al [23]. Briefly the medium was composed of IMDM (Lonza Group Ltd, Switzerland) supplemented with Fetal Bovine Serum (FBS 20% v/v, Sigma-Aldrich, St Louis, MO, USA), detoxified Human Serum Albumin (HSA 25%, Baxter International Inc., Deerfield, IL, USA), human-Stem Cell Factor (100 ng/mL h-SCF, Amgen, Thousand Oaks, CA), human-Erythropoietin (h-EPO 5 UI/mL, NeoRecormon, Roche Diagnostics, Penzberg, Germany), human Interleukin-3 (hIL-3, 1 ng/mL, Biosource, San Jose, CA, USA), L-Glutamine (L-Glu, 200 mM, Euroclone SPA, Italy), antibiotics (10,000 units/mL Penicillin G sodium, 10,000 units/mL Streptomycin sulfate and 25 μg/mL Fungizone, PSF, Lonza Group Ltd), β-Mercaptoetanol (β-Mpt 7.5×10^−5^, Sigma-Aldrich) and Poloxamer 188 (Pluronic F68, MW8400; Sigma-Aldrich), dexamethasone (DXM) and estradiol (ES) (each 10^−6^ M, Sigma-Aldrich).

The cultures were kept for up to 3 days before adding myristoylated rNef (rNef/myr) protein (50 ng/mL) or recombinant human TNF-α (10 ng/mL, PeproTech, Inc., Rock Hill, NJ, USA). Polyclonal rabbit anti-human TNF-α antibody (1 μg/mL, PeproTech, Inc.) was used in cytokine blocking experiments of Nef-treated PBMCs cultivated in HEMA culture condition. The HEMA condition without EPO was used where required by experimental procedures.

In some experiments monocytes were positively selected from total PBMCs by using CD14 magnetic beads and LS columns according to the manufacturer’s instructions (Miltenyi Biotec, Bergisch Gladbach, Germany). After isolation, cells were cultured in RPMI-1640 supplemented with 10% FBS, 1% L-Glu and 1% penicillin/streptomycin for 3 days before adding rNef/myr protein. Differentiated macrophages were obtained culturing the CD14-positive monocytes isolated by using CD14 magnetic beads (Miltenyi Biotec) in the presence of recombinant human Macrophage-Colony Stimulating Factor (M-CSF, 10 ng/mL, PeproTech, Inc.) or recombinant human Granulocyte Macrophage-Colony Stimulating Factor (GM-CSF, 50 ng/mL, R&D System, Minneapolis, MN, USA) for 5 days before adding rNef/myr protein.

### Flow Cytometry Analysis and Cell Sorting

For each sample, 1×10^5^ cells were suspended in Ca^2+^Mg^2+^-free Phosphate Buffered Saline (PBS), supplemented with 0.5% BSA, and labeled with the following anti-human antibodies: AlloPhycoCyanin (APC)-H7-conjugated CD14, Fluorescein IsoThioCyanate (FITC)- or APC-conjugated CD36 (anti-thrombospondin receptor), phycoerythrin (PE)-conjugated CD86, PE-conjugated CD206, APC-conjugated CD68, FITC-conjugated CD11c (all from BD Biosciences, Erembodegem, Belgium), PE-conjugated Toll Like Receptor-2 and 4 (TLR-2 and TLR-4, Serotec, Düsseldorf, Germany), or appropriate isotype controls. All the antibodies were incubated at the concentration of 1 μg/10^6^ cells for 30 min in the dark on ice unless otherwise advised by manufacturers. Dead cells were excluded by Sytox Blue staining (1 μM, Molecular Probes, Carlsband, CA, USA). Intracytoplasmic staining of CD68 was performed by using BD Cytofix/Cytoperm Kit (BD Biosciences) and dead cells were excluded from the analyses by Fixable Viability Dye eFluor 780 staining (eBioscience, San Diego, CA, USA). For lymphocyte and MDM purification, cells were isolated from the culture bulk by cell sorting on the basis of their forward scatter. The purity of sorted population was found >95% after reanalysis.

Stained cells were analyzed or sorted by using a BD FACSAria (BD Biosciences), equipped with three lasers (488 nm, 635 nm and 407 nm), and the results were analyzed by BD FACSDiva Software version 6.1.3 (BD Biosciences) or FlowJo Software version 7.6.1 (Tree Star, Inc., Ashland, OR, USA).

### Preparation of Recombinant Proteins

The rNef was obtained as His6-tagged fusion protein as previously described [22]. The *nef* gene from NL4-3 HIV-1 strain was amplified by PCR (Polymerase Chain Reaction) band cloned in frame with the His6 tag into the 5′-BamHI/3′-SalI sites of pQE 30 vector (Qiagen, Chatsworth, CA, USA). rNef was purified from IPTG (isopropyl *β*-D-thiogalactoside)-induced bacterial lysates in an 8 M urea buffer using Ni^2+^-nitrilotriacetate resin (Qiagen) according to the manufacturer’s instructions. rNef was eluted with 250 mM imidazole and each fraction was analyzed by SDS/PAGE (12% polyacrylamide). rNef-containing fractions were pooled and extensively dialyzed against 1x PBS to completely remove urea. rNef/myr proteins were prepared as previously described [24]. All recombinant protein preparations were scored as negative for the presence of bacterial endotoxin by using the Lymulus Amaebocyte Lysate assay (LAL test, BioWhittaker, Walkersville, MD, USA). In some experiments we used a recombinant myristoylated wild type HIV-1 Nef protein (myr2-210, C210S, SF2 strain, cat. PR-382) purchased from Bioscience (Jena, Germany).

To exclude possible signaling effects due to residual LPS traces in Nef preparations, experiments were performed in the presence of 10 μg/mL of polymyxin B (Sigma-Aldrich), a cationic antibiotic that binds to the lipid A portion of bacterial LPS [25] or by using rNef boiled at 100°C for 10 min.

### Virus Preparation and Infection

Preparations of NL4-3 HIV-1 and its derivative defective for *nef* expression (Δ*Nef*) pseudotyped with vesicular stomatitis virus (VSV-G) envelope glycoprotein were previously described [26]. Virus preparations were titrated by measuring HIV-1 CAp24 contents by quantitative enzyme-linked immunosorbent assay (Innotest HIV Antigen mAb, Innogenetics N.V., Ghent, Belgium). Infections of 5-day-old MDMs with pseudotyped HIV-1 were carried out by spinoculation at 400 g for 30 min at room temperature using 50 ng CAp24 equivalent of (VSV-G) HIV-1/10^5^ cells, followed by virus adsorption for 3 h at 37°C and addition of complete medium. After 24 and 48 h the percentages of cells expressing intracytoplasmic HIV-1 Gag-related products were evaluated by FACS analysis after permeabilization with Cytofix/Cytoperm solutions (BD biosciences) for 20 min at 4°C and labeling with 1/50 dilution of KC57-RD1 phycoerythrin (PE)-conjugated anti HIV-1 Gag CAp24 KC-57 MAb (Coulter Corp., Hialeah, FL, USA) for 1 h at 4°C.

### Quantitative Real Time-PCR

Total RNA was extracted from 10^6^ cells with the RNeasy RNA extraction kit (Qiagen). Briefly, RNA was treated with recombinant DNase I (Roche, Monza, Italy) 2 times for 1 h at 37°C each time, followed by RNA clean-up procedure according to the RNeasy kit protocol. Specifically, 1 μg of the RNA was used to synthesize cDNA by employing the Reverse Transcription (RT) System kit (Promega, Madison, WI, USA). An aliquot (2 μL) of cDNA was amplified using the oligonucleotide primers derived from the CD36 cDNA sequence [27]: forward 5′-TCAGCAAATGCAAAGAAGGGAGAC-3′and reverse 5′-GGTTGACCTGCAGCCGTTTTG-3′. The RT reaction was normalized by amplifying samples for glyceraldehyde-3-phosphate dehydrogenase (GAPDH) as house-keeping gene. CD36 primers were purchased from M-Medical-Genenco (Cornaredo, Italy) and 10× QuantiTect primer Assay Mix for GAPDH was purchased by Qiagen. RT-PCR was performed by employing the SYBR Green RT-PCR kit (Qiagen) and the Applied Biosystems 7500 Real-Time PCR System (Applied Biosystems, Carlsbad, CA, USA). Mix for each PCR point was: 12.5 μL of SYBR Green mix +9.5 μL of distilled water +2 μL of cDNA +1 μL primer mix (20 nM of each primer). Reactions were led at 95°C for 1 min, 60°C for 30 min 72°C for 1 min, for 40 cycles. Data were collected during every elongation step (72°C) and during final ramping (to control specificity), and analyzed by employing the Applied Biosystems 7500 SDS software (Applied Biosystems) using the 2^−DDCt^ method.

### Plasma Low-Density Lipoprotein (LDL) Isolation

LDLs (1.019–1.063 g/mL) were isolated by density gradient ultracentrifugation in vertical rotor as previously described [28] from pooled fresh plasma of healthy volunteers provided by Transfusional Center of Policlinico Umberto I, Sapienza University, Rome, Italy. The protein content was measured by Lowry’s method using BSA as standard. Neutral Red assay was used to assess the cytotoxicity of different LDL concentrations (25–200 mg/L) and 25 mg/L of protein concentration has been chosen to perform our experiments.

### LDL Uptake Assay

Measurements of cell oxLDL uptake were performed with fluorescence labeled lipoproteins. One milliliter of LDL (0.5 g/L) was incubated with 12 μL 1,10-Dioctadecyl-3,3,30,30-tetramethylindo-carbocyanine perchlorate (DiI) (2 mg/mL DMSO, Sigma-Aldrich) and 10 μM CuSO_4_ for 3 h as previously described [29]. Labeled oxLDL were extensively dialyzed with a centrifugal filter device (Millipore, Bedford, MA, USA) with a molecular weight cut-off of 5000 at 4°C, and sterilized through 0.22 μm filters (Millipore). Relative electrophoretic mobility of DiI-labeled oxLDL was 1.8±0.1. The degree of LDL oxidation was checked by determining the Thiobarbituric Acid Reactive Substances (TBARS) content according to Yagi [30]. The TBARS content of oxLDL was 45±7 nmol malondialdehyde equivalent/mg LDL protein. DiI-oxLDL (0.025 mg/mL of culture medium) were incubated with the cells (0.5×10^6^ cells) for 15, 30, 60 and 120 min. Cells were resuspended in PBS containing Propidium Iodide (PI) to exclude dead cells, and then kept on ice before measuring fluorescence levels with a BD FACSAria (BD Biosciences). Fluorescence levels were normalized to cells autofluorescence.

### Phagocytosis Assay

The phagocytic function was evaluated and quantified in PBMCs by following the uptake of FITC-labeled beads (Fluoresbrite BB Carboxylate Microspheres 0.50 μm, Polysciences, Inc. Eppelheim, Germany) or the internalization of Salmonella Salp572^ФIS^ strain producing the green fluorescent protein (GFP-*Salmonella tiphymurium*) [31]. In detail, for the assay performed with FITC-labeled beads, cells cultivated in HEMA w/o EPO were incubated for 30 min at room temperature with the beads at 1/5 ratio (cells/beads), washed and suspended in PBS. Dead cells were excluded from the analysis by Sytox Blue staining (Molecular Probes). To study the internalization of GFP-*Salmonella tiphymurium* cells were incubated under shacking at 37°C with bacteria for 30 min at 1/5 ratio (cells/bacteria) followed by 2 h incubation in the presence of 100 μg/mL of gentamicin (Sigma-Aldrich). Cells were washed twice in PBS and fixed in 4% paraformaldehyde (Sigma-Aldrich) in PBS. To evaluate the involvement of CD36 in the phagocytic process, cells were pre-incubated for 20 min at 37°C with mouse monoclonal anti-CD36 antibody (1 μg/10^6^ cells, FA6-152, Abcam Inc., Cambridge, MA, USA) and then incubated with beads or bacteria.

The percentage of phagocytic cells was evaluated in MDMs by flow cytometry comparing fluorescence intensity of cells that incorporate particles to the cell autofluorescence.

### TNF-α Release

PBMCs were cultivated at concentration of 5×10^5^ cells/mL for three days in HEMA condition, afterward for additional 3 days in the presence of rNef/myr. Detection of TNF-α in supernatants of HEMA-derived MDMs was performed through Human TNF-alpha Quantikine ELISA Kit from R&D System following the manufacturer’s recommendations.

### TNF-α Bioassay

TNF-α-induced cell cytotoxicity and neutralizing activity of polyclonal rabbit anti-human TNF-α antibody were measured on WEHI 164 clone 13 cells (American Type Culture Collection, ATCC; CRL-1751) [32] by a colorimetric assay using MTT originally developed by Mosmann [33]. The WEHI 164 cell line was kept in our laboratory by propagation in RPMI 1640 (Cat. No. R-6504; Sigma-Aldrich) supplemented with 10% FBS (Sigma-Aldrich), 2 mM L-Glutamine (Euroclone SPA), antibiotics (10,000 units/mL Penicillin G sodium, 10,000 units/mL Streptomycin sulfate and 25 μg/mL Fungizone, PSF, Lonza Group Ltd) and were cultured at 37°C in a humidified atmosphere with 5% CO^2^ in air. The MTT method is based on the ability of cells to convert soluble MTT into an insoluble formazan. Briefly, cells were pre-incubated with 1 mg/mL of the transcription blocker actinomycin D (Sigma-Aldrich) for 2 h. Afterwards, cells were plated in triplicate in 96-well microtitre flat-bottomed plates (1×10^5^ cells/100 μl/well) both in absence or in presence of increasing rhTNF-α concentrations or in presence of both anti-human TNF-α antibody and rhTNF-α and incubated for 24 h. Ten microliters of 5 mg/mL of MTT were then added to each well and incubated for additional 4 h. The solution was then removed and formazan salts dissolved with Sorensen’s Glycine Buffer (0.1 M Glycine plus 0.1 M NaCl in PBS). The optical densities were measured at 540 nm with reference wavelength 690 nm in a Victor3 Multilabel reader (PerkinElmer Inc, Waltham, MA, USA) All treatments were performed at 37°C in a humidified atmosphere with 5% CO_2_ air.

### Statistical Analysis

Data are presented as means ± standard deviation (S.D.). Statistical analysis was performed according to non-parametric Mann-Whitney *U* Test by using GraphPad Prism Software version 5.03 (GraphPad Software, Inc, La Jolla, CA, USA); *p*-value <0.05 was considered significant.

## Results

### rNef/myr Downregulates CD36 Expression on Human Monocytes

**Figure 1:**
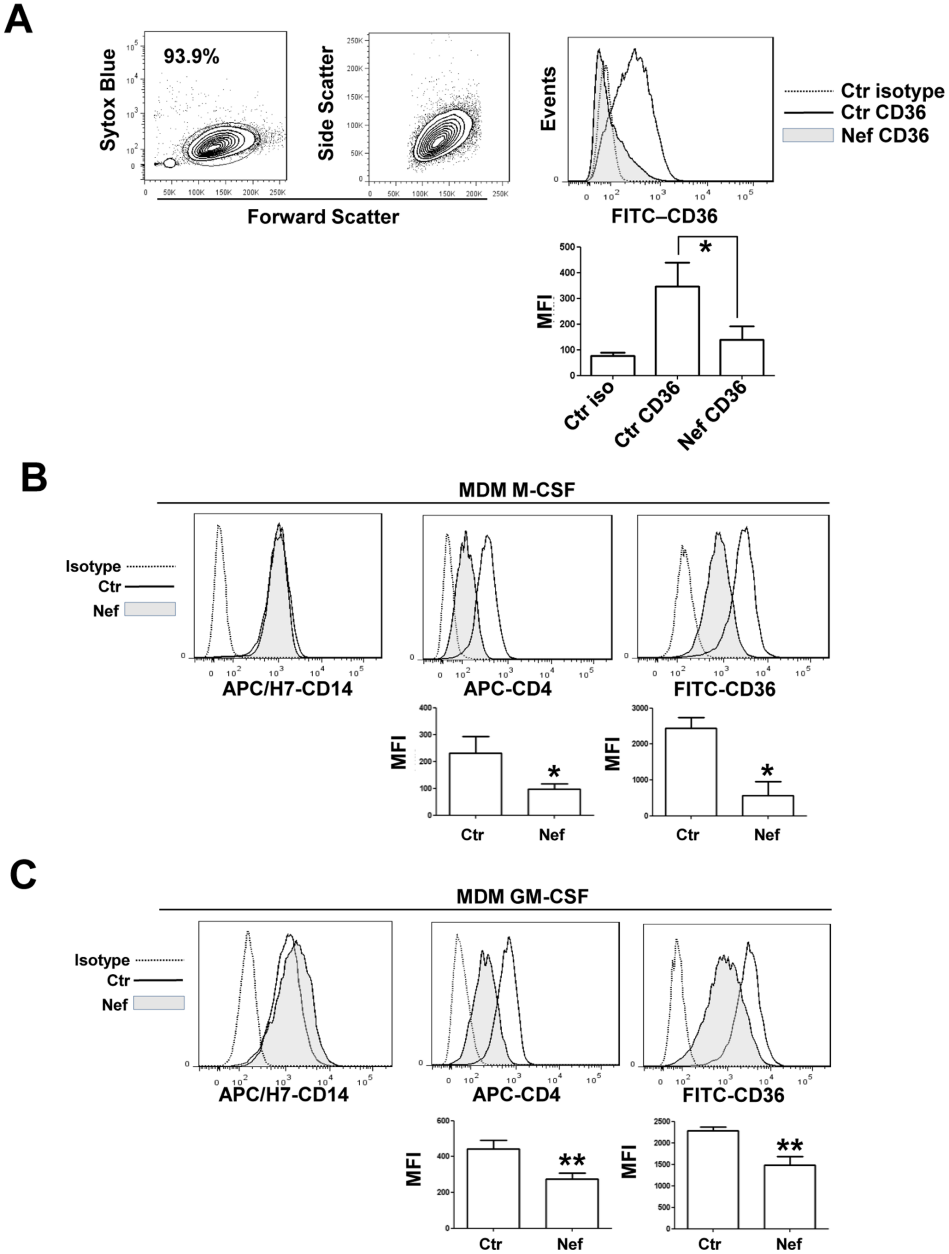
Nef induces downregulation of CD36 expression in human PBMC-derived monocytes. Purified monocytes evaluated for CD14 expression (data not shown) were cultured in presence 50 ng/mL of rNef/myr for three days. (A) Representative dot plots and histogram of monocytes analyzed by flow cytometry. The dot plot on the center shows the forward and side scatter of monocytes. On the left is shown the viability of cells by using SYTOX Blue dead cell stain. CD36 expression, shown in the histogram, was analyzed by using FITC-conjugated anti-CD36 antibody and the fluorescence intensity in Nef-treated (solid grey histogram) was compared to untreated (solid line) cells. Matched isotype (dotted line) was used as control of non-specific fluorescence signals. (A, below the histogram) The column bar graph represents the MFI of cells stained with matched isotype control (Ctr iso), cells stained with FITC-conjugated anti-CD36 antibody (Ctr CD36), and Nef-treated cells stained with FITC-conjugated anti-CD36 antibody (Nef CD36). The results (mean ± standard deviation) are representative of five independent experiments (*p<0.05). In B and C representative histograms of M-CSF and GM-CSF differentiated MDMs evaluated for CD14, CD4 and CD36 expression are shown. Cells were isolated by using CD14 magnetic beads (Miltenyi Biotec) and cultured in presence of human M-CSF (10 ng/mL) or GM-CSF (50 ng/mL) for 5 days before adding rNef/myr protein. CD14, CD4 and CD36 expression was analyzed by using appropriate fluorochrome-conjugated antibodies and the fluorescence intensities in Nef-treated (solid grey histogram) were compared to untreated (solid line) cells. Matched isotype (dotted line) was used as control of non-specific fluorescence signals. (B and C, below the histograms) The column bar graphs show the MFI of untreated cells (Ctr) and Nef-treated cells (Nef) stained with APC- and FITC-conjugated anti-CD4 and CD36 antibodies, respectively. SYTOX Blue has been used to exclude dead cells from the analyses. The results (mean ± standard deviation) are representative of four (MDM M-CSF) and six (MDM GM-CSF) independent experiments (*p<0.05, (**p<0.01).

CD36 represents a pattern recognition receptor implicated in a wide variety of normal and pathologic biological functions and mediates the uptake of various bacterial pathogens. Here we examined the effect of soluble Nef protein on human monocyte/macrophage CD36 expression in order to identify a new viral mechanism directed to impairing phagocytosis and other macrophage functions. PBMC-derived monocytes from healthy donors were obtained as described in Materials and Methods. The purified monocytes evaluated for CD14 expression (data not shown) were cultured in the presence of 50 ng/mL of rNef/myr for additional 3 days and analyzed for CD36 expression. The flow cytometry analysis (Fig. 1A) shows a dramatic downregulation of CD36 expression. This decreased expression results highly significant only at 3 days from Nef addition to the cell culture while at 1 or 2 days the CD36 reduction appears not significant, probable as a consequence of cell culture system variability (not shown). We also evaluated CD36 modulation in MDMs by culturing CD14 positive cells for 5 days in the presence of M-CSF or GM-CSF to induce macrophage differentiation. Cells were treated with rNef/myr for additional 3 days and analyzed by flow cytometry. In figures 1B and 1C the CD14, CD4 and CD36 expression levels, measured in M-CSF and GM-CSF differentiated MDMs are shown. In these cells, 3 days treatment with rNef/myr induces a significant downregulation of CD36 expression in both culture conditions. As control of Nef activity we also evaluated the CD4, a well-known receptor whose surface expression is modulated by the HIV-1 Nef protein. As expected rNef/myr induced a significant decrease in CD4 expression in both M-CSF and GM-CSF differentiated MDMs. Interesting rNef/myr does not modify the expression levels of CD14.

### rNef/myr Selectively Regulates CD36 Expression on PBMC-derived Macrophage-like Cells Cultivated in HEMA Condition

In our laboratory we developed the HEMA culture system [23] in order to obtain a massive in vitro expansion of human erythroid cells starting from total PBMCs. The population identified in culture expanded for three and six days is mainly represented by erythroblasts at different stage of differentiation, lymphocytes, and monocyte/macrophage cells [34].

**Figure 2:**
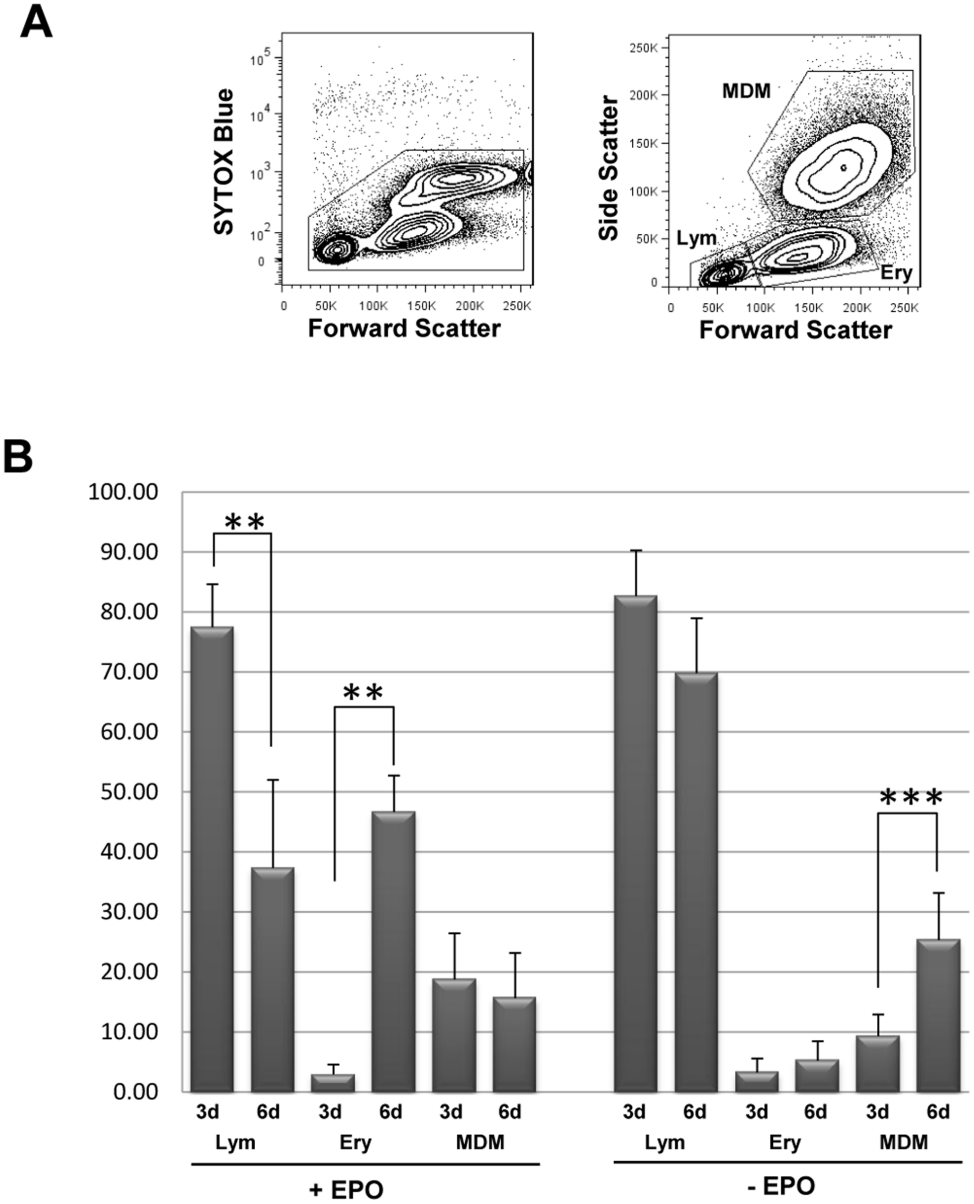
PBMCs cultivated under HEMA cell culture conditions produce three main populations. PBMCs were cultivated in HEMA condition (see Materials and Methods) for three and six days. (A) Representative dot plots PBMCs analyzed at six days in HEMA condition by flow cytometry. The dot plot on the left shows the viability of cells by using SYTOX Blue dead cell stain. The dot plot on the right shows the three main populations identified by characteristic forward and side scatter: a lymphocyte gate (Lym), erythroblast gate (Ery) and MDMs. (B) PBMCs have been cultivated in HEMA culture condition in presence or absence of EPO. The relative percentages of the three populations (Lym, Ery, MDMs) at three (3d) and six (6d) days in HEMA (+/− EPO) culture are presented in the histogram. The results (mean ± standard deviation) are representative of six (HEMA condition) and twelve (HEMA w/o EPO condition) independent experiments (**p<0.01, ***p<0.005).

As shown in Fig. 2A, PBMCs cultivated under HEMA cell culture conditions produce three main populations with distinctive forward and side scatter profile (Fig. 2A): a lymphocyte-like population (Lym gate 37.8±14.7%), erythroblast cells (Ery gate 46.7±6.1%), and a MDMs population (MDMs gate 15.7±7.5%). Therefore, six days of complete HEMA culture condition allowed us to analyze the effects of Nef on CD36 expression in different cell lineages at the same time, i.e. Ery and MDM cells. Longer time of culture in the presence of EPO determines a higher expansion of the Ery population with a dramatic decrease in MDM population. On the other hand, removal of EPO from the HEMA culture condition determines a strong inhibition of erythroblasts expansion (5.5±3.2) with a relative increase in MDMs (27.6±8.4); this is a useful condition for analysis aimed at studying the MDM population (Fig. 2B).

**Figure 3:**
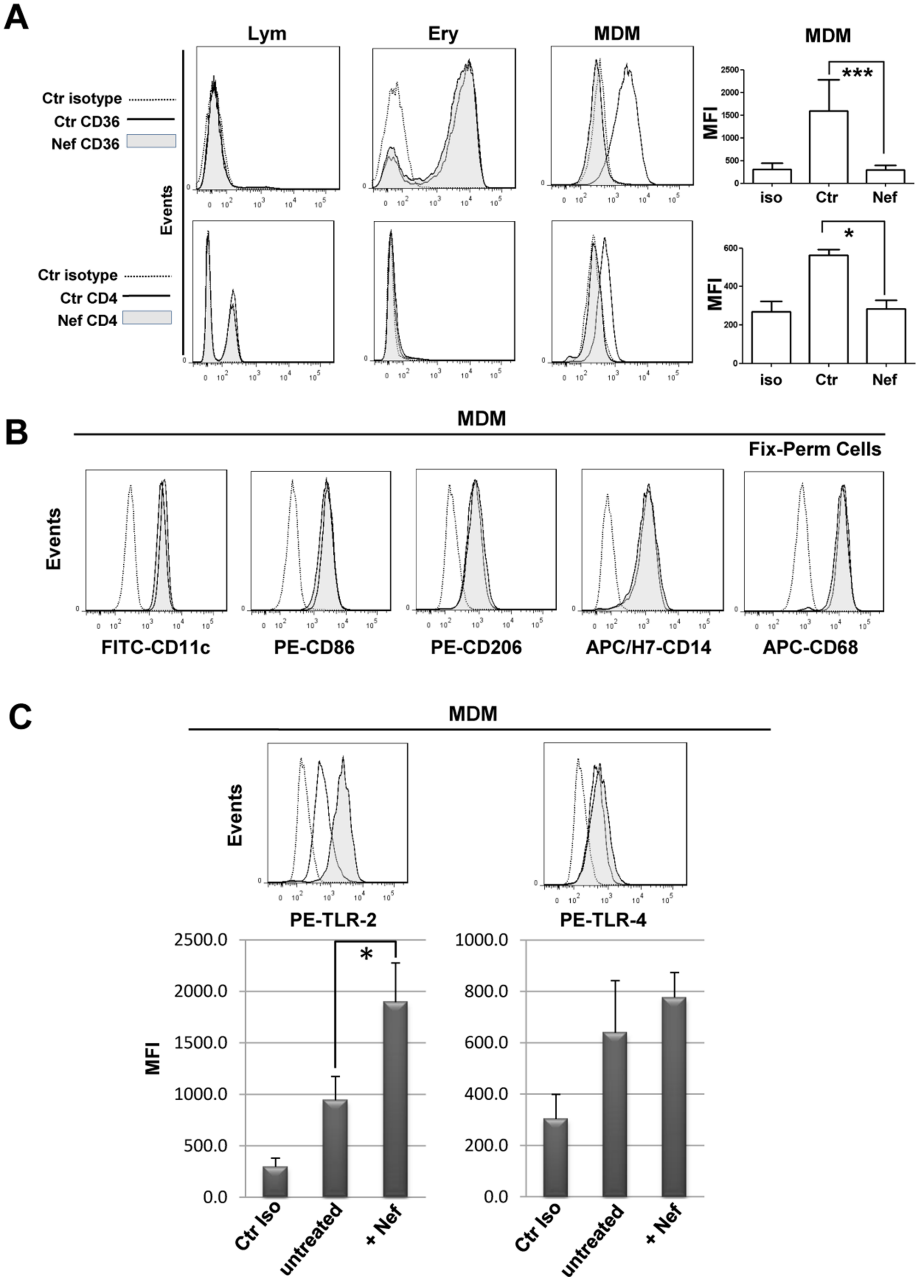
CD36 expression on MDM cells cultivated in HEMA condition treated with recombinant Nef. PBMCs were cultivated in HEMA condition for three days and for additional three days in presence of 50/mL rNef/myr (A) Representative histograms of the three populations (Lym, Ery, MDMs) analyzed for CD36 and CD4 expression by flow cytometry at six days expansion. The respective populations were identified as described in figure 2A and the fluorescence intensities in Nef-treated (solid grey histogram) compared to untreated (solid line) cells are shown. Matched isotype (dotted line) was used as control of non-specific fluorescence signals. In the column bar graphs on the right of the histograms are presented the MFI of cells stained with matched isotype control (iso), untreated cells (Ctr) and Nef-treated cells (Nef) stained with FITC- or APC-conjugated anti-CD36 and CD4 antibodies. The results are representative of ten (CD36) and four (CD4) independent experiments. MDMs were analyzed by flow cytometry for the expression of several specific markers, i.e. CD14, CD11c, CD86, CD68 and CD206. Representative histograms are shown in (B) and the fluorescence intensities of respective antibodies in Nef-treated (solid grey histogram) were compared to untreated (solid line) cells. The results are representative of five independent experiments. (C) Representative histograms of PBMC-derived MDMs analyzed by flow cytometry for the expression of TLR2 and TLR4. The MFI of Nef-treated (+ Nef) compared to untreated (untreated) cells was reported in the respective histograms. The results are representative of five independent experiments (*p<0.05). Matched isotype (dotted line or Ctr iso) was used as control of non-specific fluorescence signals. SYTOX Blue was used to exclude dead cells in all the experiments presented in this figure.

The PBMCs were cultivated in HEMA culture condition without EPO for three days, afterward for additional 3 days in the presence of rNef/myr and analyzed by flow cytometry for the expression of several MDM markers. As shown in Fig. 3A, the treatment with rNef/myr induces a dramatic reduction of CD36 surface expression only on MDMs. Furthermore, a significant reduction in CD4 expression is observed, as expected by the recognized activities of Nef protein. Moreover, in MDMs, rNef/myr does not modify expression levels of CD14, CD11c, CD86, CD68 and CD206 (Fig. 3B). Interestingly, rNef/myr treatment does not down-regulate CD36 expression in Ery cells and CD4 in Lym cells (Fig. 3A). In synthesis, these results indicate that Nef specifically affects CD36 and CD4 expressions while does not modify the expression of other MDM markers. Furthermore, the lack of effect on CD36 and CD4 expressions in Ery and Lym cells suggests a cell specific response still to be clarified, although it is probably caused by the incapacity of erythroblasts and lymphocytes to take up the Nef protein efficiently. We also evaluated the expression of Toll-like receptor 2 and 4 (TLR2, TLR4), the type-I transmembrane proteins crucial in the recognition of pathogen-associated molecular patterns [35] and in the interaction with CD36 in inflammation and phagocytosis exerted by the innate immune system [36], [37]. Differently by CD36, TLR4 is not inhibited in cells treated with rNef/myr while the TLR2 expression significantly increases (Fig. 3C). It should be underlined that the two different culture conditions, with or without EPO, do not affect the phenotypic profile of MDMs and, most important, the rNef/myr-dependent CD36 downregulation (not shown).

### Specificity of Nef-induced CD36 Downregulation

**Figure 4:**
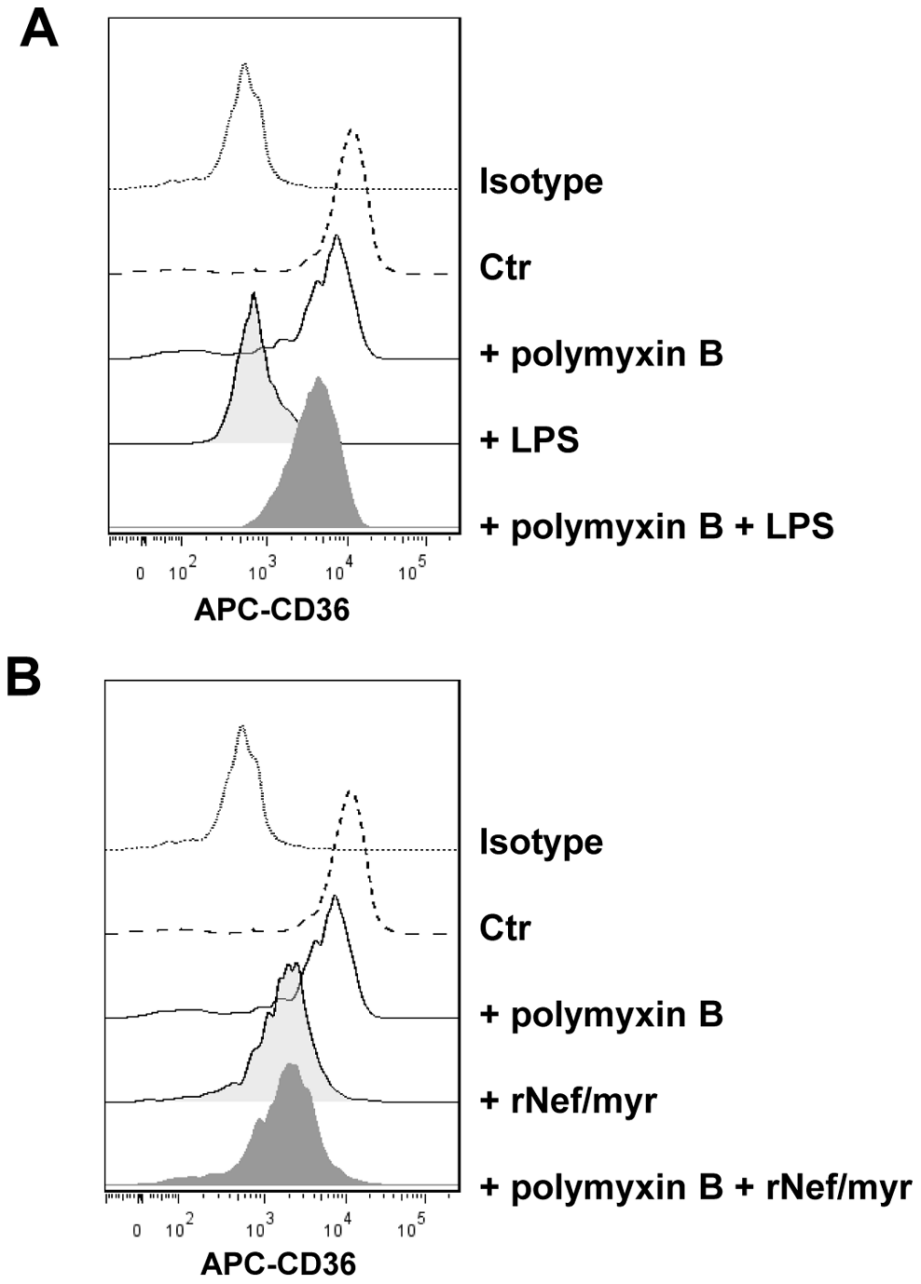
CD36 expression on MDM cells cultivated in HEMA condition in presence of the LPS inhibitor polymyxin B. PBMCs were cultivated in HEMA condition for three days followed by additional three days in presence of 50/mL rNef/myr, 100 ng/mL LPS or 10 μg/ml polymyxin B. In some cultures, polymyxin B was added 15 min before the Nef and LPS treatment. (A) Representative histogram of MDMs analyzed by flow cytometry for the expression of CD36. The histogram on the right shows the respective treatment with LPS, polymyxin B and polymyxin B+LPS. The fluorescence intensities were compared to untreated cells (Ctr). In (B) similar analysis by replacing LPS with rNef/myr is shown. Both in (A) and (B) matched isotypes (Isotype) were used as control of non-specific fluorescence signals. SYTOX Blue was used to exclude dead cells. The results are representative of three independent experiments.

As already described [38], [39] the bacteria cell wall component, lipopolysaccharide (LPS) induces CD36 downregulation. Thus, we evaluated specifically the effect of the LPS inhibitor polymyxin B on LPS- and Nef-mediated CD36 regulation although all the used batches of purified rNef protein preparations were tested with assay to exclude LPS contamination (see Material and Methods). As expected, induction of cell culture with LPS reduces the levels of CD36 membrane expression whereas pre-treatment with polymyxin B completely counteracts the LPS effect (Fig. 4A). Conversely, the polymyxin B pre-treatment does not influence Nef-dependent CD36 downregulation (Fig. 4B). These results definitely exclude a potential contribution of contaminant LPS to the Nef-mediated CD36 downregulation.

### Nef Myristoylation is Required for Stronger Activity

**Figure 5:**
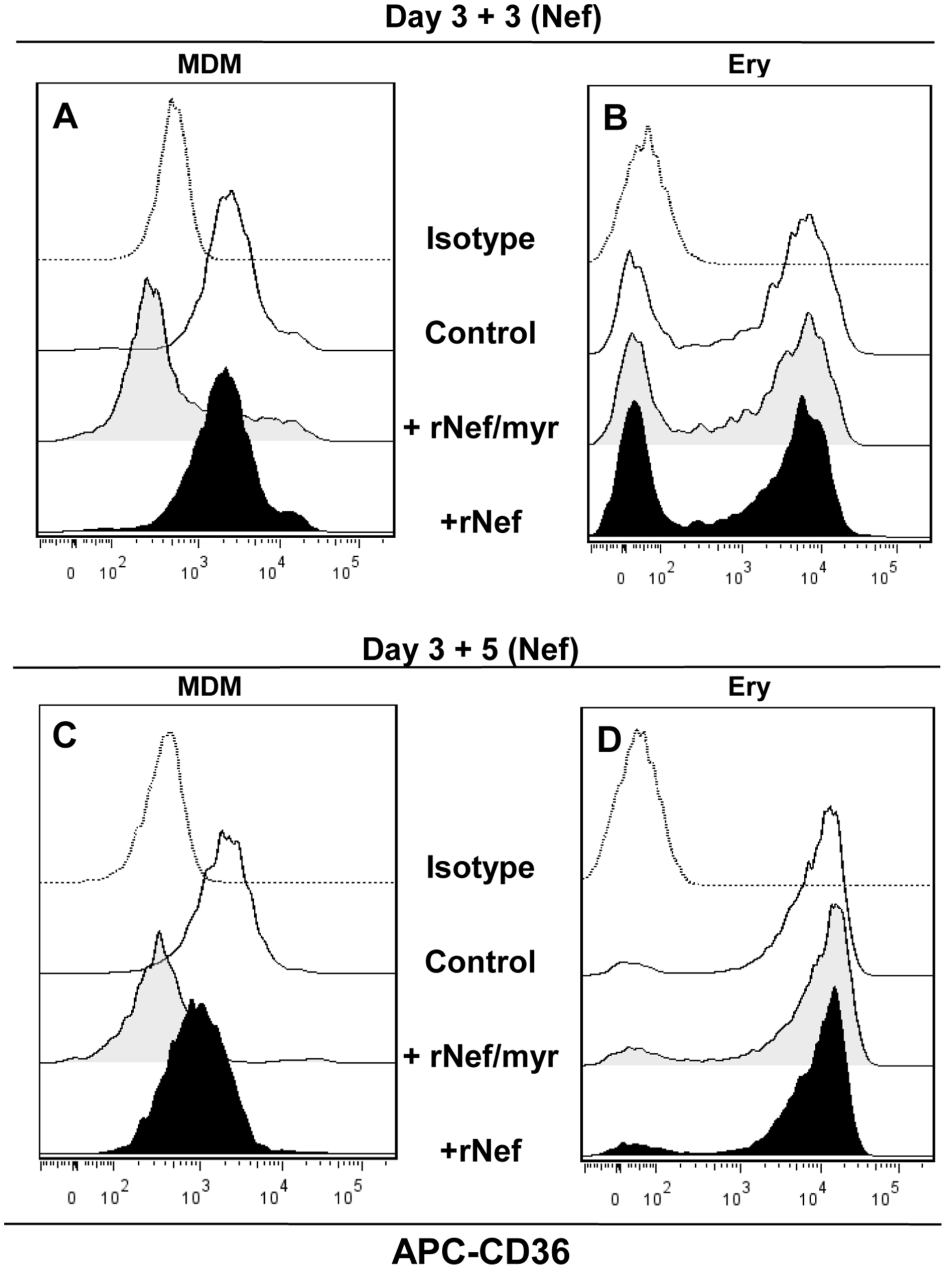
Comparison of myristoylated and non-myristoylated Nef activity on CD36 expressed by MDM and erythroblast cells cultivated in HEMA condition. PBMCs were cultivated in HEMA condition for three days followed by additional three or five days in presence of 50/mL rNef/myr and rNef. Representative histogram of MDM (A) and Ery (B) cells analyzed by flow cytometry for the expression of CD36 after three days of Nef treatment while in (C) and (D) MDM and Ery cells analyzed after five days of Nef treatment are shown. The fluorescence intensities were compared to untreated cells (Ctr). In all experiments matched isotypes (Isotype) were used as control of non-specific fluorescence signals. SYTOX Blue was used to exclude dead cells. The results are representative of three independent experiments.

Asztalos et al [40] have demonstrated that recombinant myristoylated HIV-1 Nef added to the extracellular milieu of cultured human MDMs suppresses cholesterol efflux in a dose dependent manner whereas non-myristoylated Nef (rNef) is ineffective. To verify a similar behavior on CD36 expression we compared the activity of rNef/myr or rNef on mononuclear cells cultivated as above described and incubated with both the rNef proteins for three days or prolonged time (five days). As expected, rNef/myr addition on macrophage-like cells induces a dramatic reduction of CD36 expression at either, three and five days of treatment. Instead rNef was able to slightly reduce CD36 expression only at five days of treatment (Fig. 5A, C); no effect was observed on Erythroblast cells (Fig. 5B, D), as previously reported (Fig. 3A). The mechanism underlying the different effect between rNef/myr and rNef could be ascribed to reduced cellular uptake or failure to localize in membrane of not myristoylated Nef compromising its intracellular biological responses. It is worth noting that eight days of HEMA culture induces the expression of CD36 in all erythroblast cells (Fig. 5B, D, black histogram) [34].

### CD36 is Downregulated in MDMs Infected in vitro with Nef(+)HIV-1

**Figure 6:**
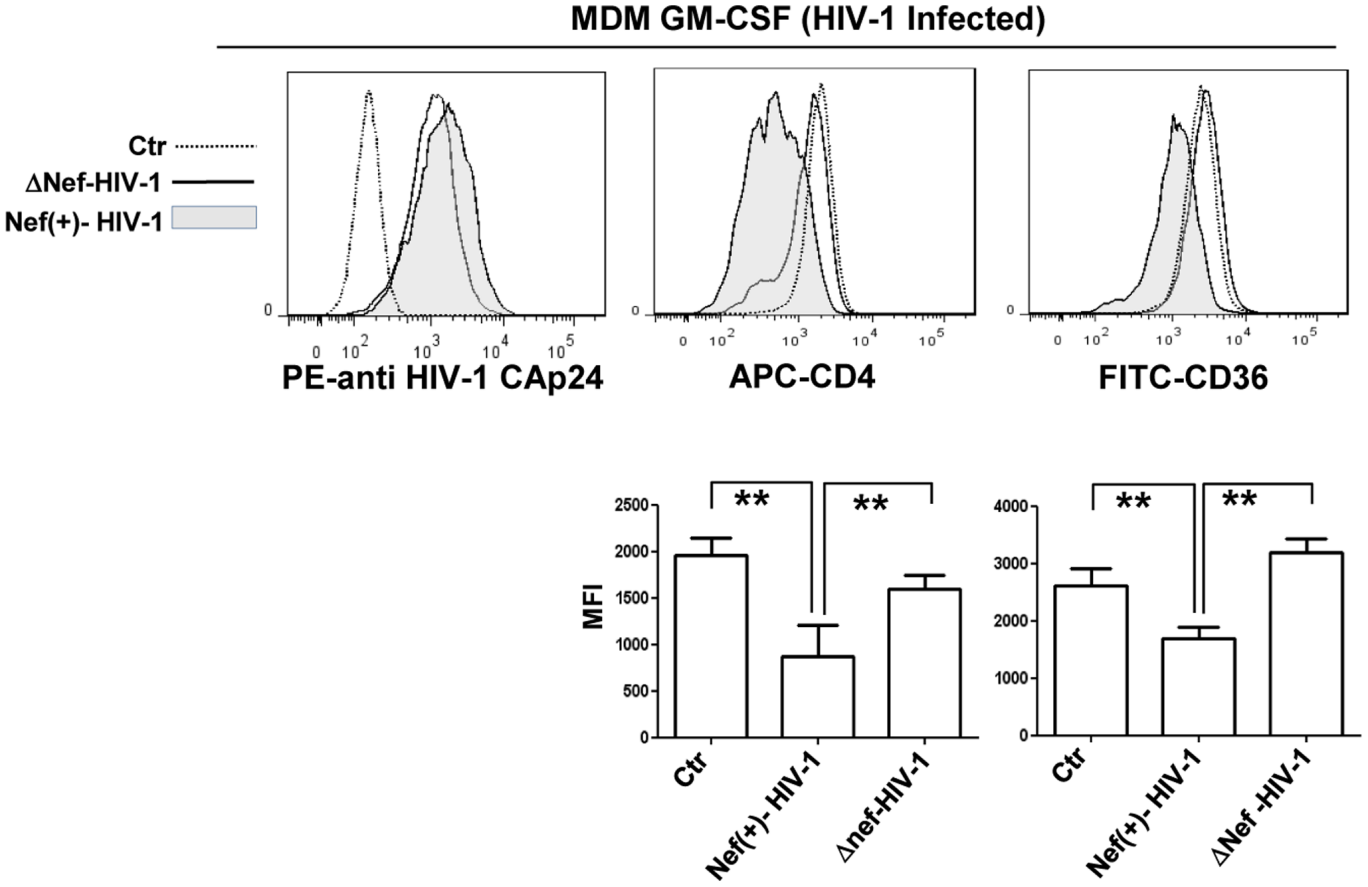
CD36 and CD4 expression in MDM GM-CSF cells infected in vitro with Nef(+)-HIV-1. MDMs differentiated in presence of GM-CSF for 5 days were infected with VSV-G pseudotyped HIV-1-expressing (Nef (+)-HIV-1) or not expressing the *nef* gene (ΔNef-HIV-1). In the left histogram the infection efficiency is shown; it is evaluated by estimating the levels of intracytoplasmic HIV-1 Gag-related products (HIV-1 CAp24) by flow cytometry analysis at 48 h postinfection. CD4 and CD36 expression was analyzed by using APC- and FICT-conjugated antibodies (center and right histogram, respectively) and the fluorescence intensities in Nef(+)-HIV-1-infected (solid grey histogram) was compared to ΔNef-HIV-1-infected (solid line) cells. Matched isotype (dotted line) was used as control of non-specific fluorescence signals. The column bar graphs, below the respective histograms, represent: the MFI of untreated cells (Ctr), and Nef(+)-HIV-1- and ΔNef-HIV-1-infected cells stained with APC-conjugated anti-CD4 and FITC-conjugated CD36 antibodies. SYTOX Blue has been used to exclude dead cells from the analyses. The results (mean ± standard deviation) are representative of four (MDM M-CSF) and six (MDM GM-CSF) independent experiments (**p<0.01).

The main target of this study is to assess the effects of soluble Nef on MDMs as autocrine/paracrine activities, although it is remarkable to verify, in a viral framework, the maintaining of Nef ability to downregulate CD36 expression in HIV-1 infected MDMs. For this purpose, GM-CFS differentiated MDMs at 6 days were infected with VSV-G pseudotyped HIV-1-expressing (Nef(+)-HIV-1) or not expressing the *nef* gene (ΔNef-HIV-1). The infection efficiency was controlled by estimating the levels of intracytoplasmic HIV-1 Gag-related products (HIV-1 CAp24) by flow cytometry analysis at 24 and 48 h post-infection. In Fig. 6 (right histogram) are shown the levels of infection at 48 h of both Nef(+)-HIV-1 and ΔNef-HIV-1 VSV-G pseudotypes and no significant differences were found between them. Similar level of HIV-1 CAp24 was observed at 24 h post-infection (not shown). CD36 expression evaluated by flow cytometry appears significantly reduced in MDMs infected with Nef(+)-HIV-1 only (Fig. 6, center histogram and right column bar graph). As control of Nef activity we evaluated CD4 expression in the same experimental condition. Nef(+)HIV-1 induces a significant decrease in CD4 expression similarly to rNef/myr (Fig. 6, left histogram and left column bar graph). These data appear consistent with those obtained in rNef/myr-treated cells and establish a stringent correlation between Nef and modulation of CD36 expression.

### Nef-dependent Downregulation of CD36 Involves RNA Transcriptional Inhibition

**Figure 7:**
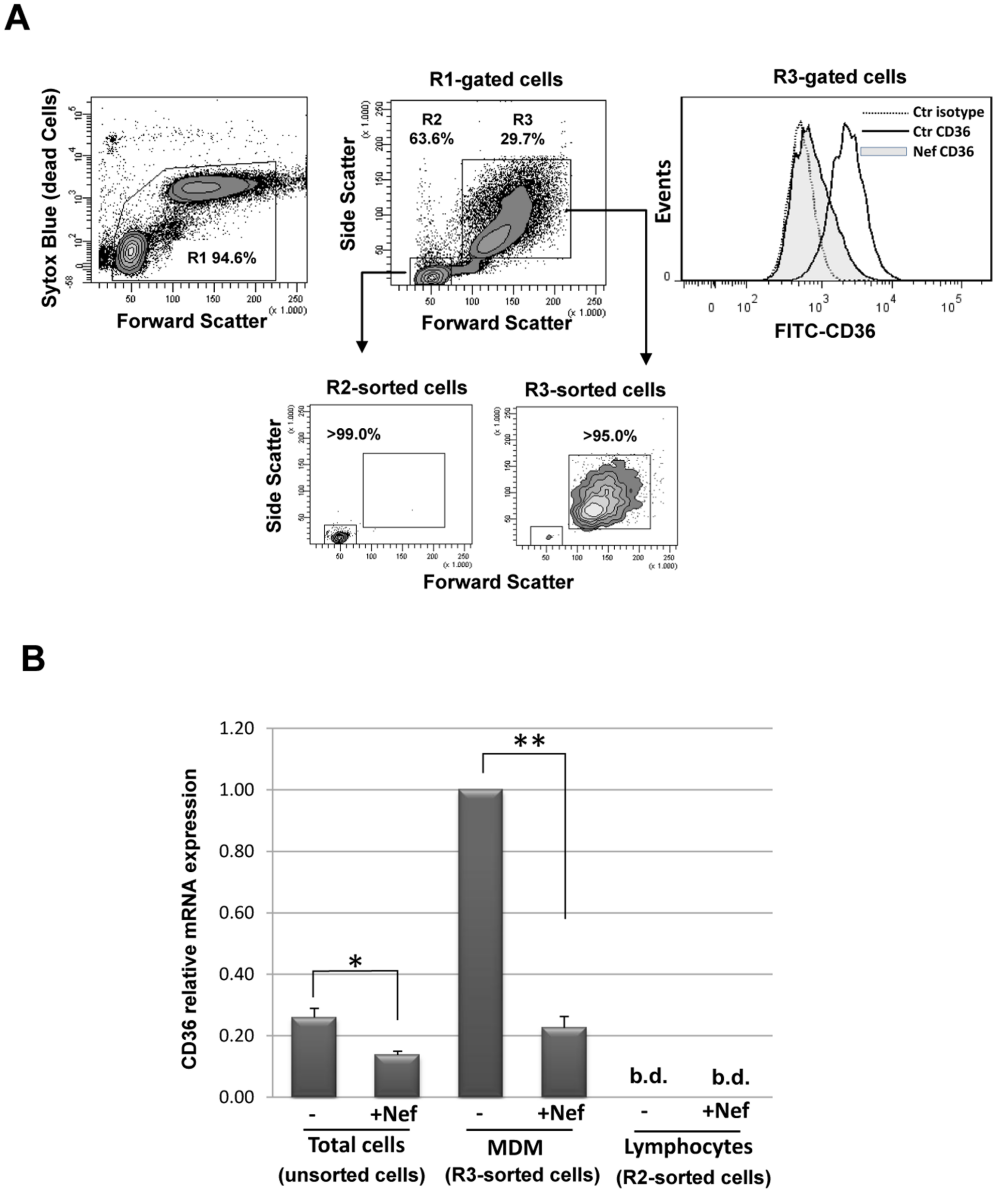
CD36 RNA transcriptional levels in Nef treated cells. PBMCs were cultivated in HEMA condition w/o EPO for three days followed by additional three days in presence of 50 ng/mL rNef/myr. (A) Representative dot plots and histogram of PBMCs analyzed by flow cytometry. The viability of cells, evaluated by SYTOX Blue dead cell stain (R1), is shown on the left dot plot. The dot plot on the center shows the forward and side scatter of expanded PBMCs (R1-gated cells). The R2 (Lym) and R3 (MDM) populations were identified as described in figure 2A. The smaller dot plots show the Lym (R2-sorted) and MDM (R3-sorted) cells separated by cell sorting and the purity was >99% and >95%, respectively. The histogram on the right shows the CD36 expression, analyzed on MDMs by using FITC-conjugated anti-CD36 antibody, and the fluorescence intensity in Nef-treated (solid grey histogram) was compared to untreated (solid line) cells. Matched isotype (dotted line) was used as control of non-specific fluorescence signals. (B) The histogram reports the CD36 relative mRNA levels expression of CD36 assessed in expanded PBMCs (Total cells), MDMs (R3-sorted cells) and Lymphocytes (R2-sorted cells) cultivated in presence or absence of Nef. RT-PCR results are normalized to the GADPH housekeeping gene. The results (mean ± standard deviation) are representative of three independent experiments (*p<0.05, **p<0.01); b.d., below of detection.

We used quantitative RT-PCR to assess whether the decrease in CD36 protein levels observed in rNef/myr treated cells is linked to mRNA transcriptional inhibition. RNA was extracted from total PBMCs cultivated under HEMA w/o EPO for three days and treated with rNef/myr for additional three days, and from the respective FACS-purified Lym and MDM cells (Fig. 7A). As shown in Fig. 7B, the treatment with rNef/myr significantly reduces the level of CD36 RNA transcript of approximately 40% in total PBMCs; an inhibition of 80% is observed in purified MDMs while no appreciable level of CD36 RNA is found in Lym cells. These results are reasonably concordant with the level change of CD36 membrane form expressed on rNef/myr-treated cells.

### Nef Inhibits Oxidized Lipoprotein (oxLDL) Uptake MDMs

**Figure 8:**
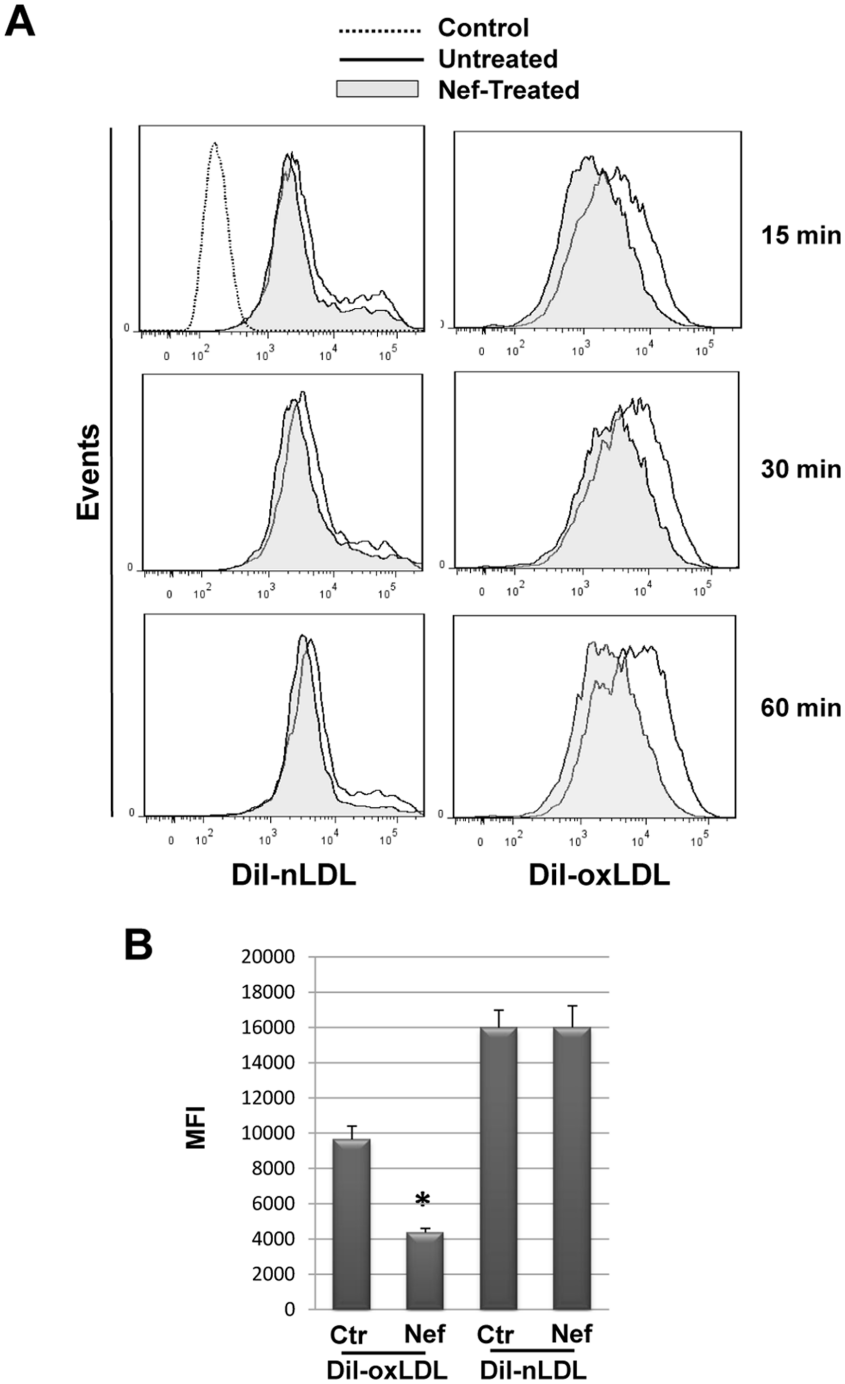
Recombinant Nef reduces oxLDL uptake in MDMs. PBMCs were cultivated in HEMA condition w/o EPO for three days followed by additional three days in presence of 50 ng/mL rNef/myr. Cells were then incubated with 25 μg/mL of DiI-nLDL or DiI-oxLDL in delipidized serum for different time (15, 30 and 60 min). (A) Representative histograms of fluorescent lipids uptake by Nef-treated (solid grey histogram) compared to untreated (solid line) cells evaluated by flow cytometry. Cells w/o DiI-conjugated lipids (dotted line) were used as control for autofluorescent signal. (B) The MFI of Nef-treated (Nef) compared to untreated (Ctr) cells at 60 min from the DiI-conjugated lipids addition were reported in the histogram. SYTOX Blue was used to exclude dead cells. The results (mean ± standard deviation) are representative of three independent experiments (*p<0.05).

The functional relevance of Nef-induced CD36 downregulation was demonstrated by investigating the capacity to internalize oxLDL by MDMs. PBMCs were cultivated under HEMA condition w/o EPO for three days and in presence of rNef/myr for 3 additional days. Nef-induced CD36-downregulation was verified by flow cytometry analysis in cells incubated with 25 μg/mL of DiI-conjugated native (DiI-nLDL) or oxidized LDL (DiI-oxLDL). In particular we observed that LDL accumulation inside the cells increases with the incubation time regardless of the lipoprotein oxidation state (Fig. 8A). However, in rNef/myr treated cells oxLDL incorporation is significantly inhibited showing at 1 h a Mean Fluorescence Intensity (MFI) of 4365±235 versus 9650±750, while nLDL accumulation is not influenced by Nef treatment (MFI of 13950±175 versus 14350±350) (Fig. 8B). No significant oxLDL incorporation by lymphocytes or erythroblasts is observed (not shown). Furthermore, longer exposure time to oxLDL determines a saturation effect with a progressive reduction of the difference in lipoprotein accumulation between untreated and Nef-treated cells (not shown). This result has not been investigated yet but suggests a possible mechanism for the LDL incorporation plateau that could involve other scavenger receptors or non-specific uptake.

### Nef Reduces Beads and Salmonella Tiphymurium Phagocytosis in MDMs

**Figure 9:**
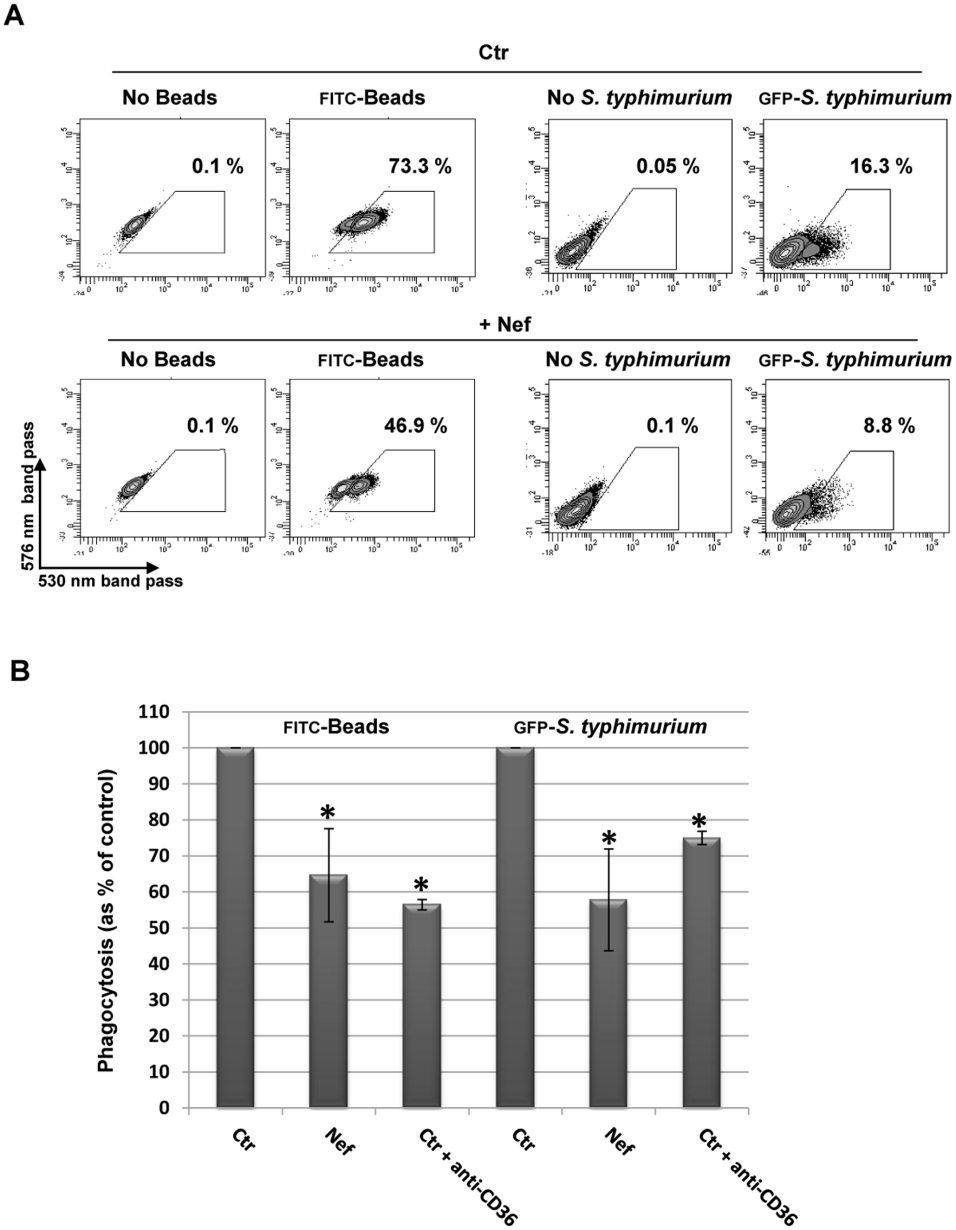
Recombinant Nef reduces phagocytosis in MDMs. PBMCs were cultivated in HEMA condition w/o EPO for three days followed by additional three days in presence of 50 ng/mL rNef/myr. Cells were then incubated with FITC-labeled beads or GFP-*S. typhimurium* for 30 min. (A) Representative dot plots of fluorescent beads and *S. typhimurium* uptake by Nef-treated (+Nef) compared to untreated (Ctr) cells evaluated by flow cytometry. Cells not incubated with beads or *S. typhimurium* were used as control for auto-fluorescent signal. The gates indicate the respective percent of phagocytosis. (B) The phagocytosis capability of Nef-treated (Nef) expressed as percent of control (Crt) is reported in the histogram. Where required by experimental procedures, control cells were pre-incubated with blocking anti-CD36 antibody for 20 min before the phagocytosis assay (Crt+anti-CD36). SYTOX Blue was used to exclude dead cells. The results (mean ± standard deviation) are representative of four independent experiments (*p<0.05).

As a pattern recognition receptor, CD36 plays an important role in phagocytosis of several non-opsonized microbial pathogens as widely described [7]–[9], [41]. In order to evaluate the impairment of other MDMs functional activities by Nef-induced CD36 downregulation, we tested the ability of Nef-treated cells to internalize FITC-conjugated microparticles (0.5 μm) and non-opsonized GFP-*Salmonella typhimurium* (Fig. 9). PBMCs were cultivated under HEMA condition w/o EPO for three days and for additional 3 days in presence of rNef/myr. CD36 downregulation was verified by flow cytometry analysis before the phagocytosis assays. Phagocytosis of microparticles and bacteria measured by flow cytometry is inhibited by Nef treatment in both cases, as shown in Fig. 9A. In order to establish the role and the level of CD36 contribution in the phagocytosis, cells were pre-incubated with blocking antibody anti-CD36 receptor for 30 min before the phagocytosis assays. The results, shown in Fig. 9B, demonstrate that CD36 is actively involved in the uptake of both microparticles and bacteria phagocytosis. Indeed the addition of CD36 blocking antibody determines a significant reduced internalization of approximately 44% and 25% of microparticles and bacteria, respectively. These data are not dissimilar from those obtained in the presence of rNef/myr (35% and 42%, respectively).

### Relationship between Nef-induced TNF-α Release and CD36 Downregulation in MDMs

**Figure 10:**
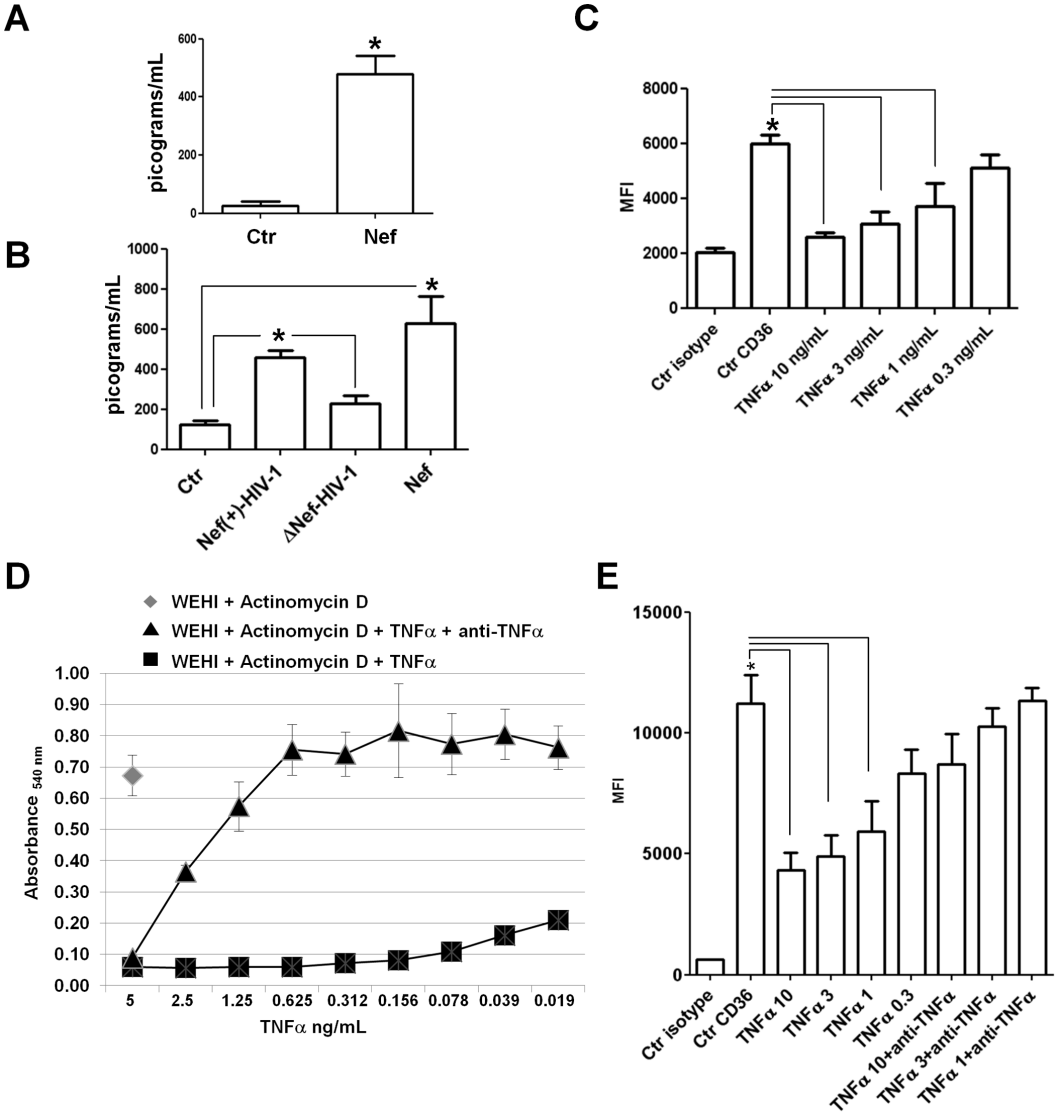
Nef induces TNF-α release and anti-human TNF-α antibody neutralizes rhTNF-α-induced CD36 downregulation. TNF-α release was measured into supernatants collected from MDM cultures in presence or absence of Nef protein, (A) The column bar graph shows the amount of TNF-α released in the medium by untreated cells (Ctr) or Nef-treated cells (Nef) derived from PBMCs cultivated in HEMA condition w/o EPO for three days and in presence of rNef/myr for additional three days. (B) The column bar graph shows the amount of TNF-α released in the medium by MDMs differentiated in presence of M-CSF (10 ng/mL) for 5 days and treated with rNef/myr or infected with VSV-G pseudotyped HIV-1-expressing or not expressing the *nef* gene. (Ctr) untreated cells, (Nef(+)-HIV-1) infected MDMs with VSV-G pseudotyped HIV-1-expressing Nef, (ΔNef-HIV-1) infected MDMs with VSV-G pseudotyped HIV-1-not expressing Nef, (Nef) rNef/myr-treated cells. The levels of the cytokine are expressed as picograms/mL. The results (mean ± standard deviation) are representative of three independent experiments (*p<0.05). (C) Cells isolated by using CD14 magnetic beads (Miltenyi Biotec) were cultured in presence of human M-CSF (10 ng/mL) for 5 days followed by additional three days in presence of different concentrations of rhTNF-α (10, 3, 1, 0.3 ng/mL). In the column bar graph are presented the MFI of untreated cells (Ctr CD36) and TNF-α-treated cells at different cytokine concentrations (TNFα 10, 3, 1, 0.3 ng/mL) stained with FITC-conjugated anti-CD36. Matched isotype (Ctr isotype) was used as control of non-specific fluorescence signals and SYTOX Blue was used to exclude dead cells. The results (mean ± standard deviation) are representative of three independent experiments (*p<0.05). (D) Measurement of cytotoxic activity of serial diluted concentrations of rhTNF-α on WEHI-164 cells by using a MTT assay. In the line graph the absorbance of multiwells containing cells pre-incubated in presence of actinomycin D (♦, 1 μg/mL) with addition of rhTNF-α alone (▪) or together with anti-human TNF-α antibody (▴) is reported. The data shown are representative of two independent experiments carried out in triplicate. (E) M-CSF-derived MDMs were treated for three days with different concentrations of rhTNF-α alone (10, 3, 1, 0.3 ng/mL) or together with anti-human TNF-α antibody (1 μg/mL). The column bar graph represent the MFI of untreated cells (Ctr CD36), TNF-α-treated cells at different cytokine concentrations (TNFα 10, 3, 1, 0.3 ng/mL) or cells incubate with both rhTNF-α and 1 μg/mL of anti-human TNF-α antibody (TNFα 10, 3, 1 ng/mL+anti TNFα) stained with FITC-conjugated anti-CD36. Matched isotype (Ctr isotype) was used as control of non-specific fluorescence signals and SYTOX Blue was used to exclude dead cells. The results (mean ± standard deviation) are representative of three independent experiments (*p<0.05).

Previous reports (17, 20–22) have demonstrated that Nef induces the release of inflammatory factors including the TNF-α in MDMs. Furthermore, Boyer et al [42] have shown that this factor was able to inhibit CD36 membrane expression and the respective mRNA transcription in human monocytes. We tested the capacity of Nef to stimulate the release of TNF-α by MDMs differentiated in HEMA culture conditions w/o EPO and in M-CSF-differentiated MDMs treated with rNef/myr or infected *in vitro* with VSV-G pseudotyped HIV-1-expressing (Nef(+)-HIV-1) or not expressing the *nef* gene (ΔNef-HIV-1). The results shown in Fig. 10A and B demonstrate a significant increment of TNF-α release in all the culture conditions treated with Nef (see also Table 1).

**Table 1:** TNF-α released by MDMs.

	HEMA-differentiated MDMs		M-CSF-differentiated MDMs			
	Ctr	rNef/myr	Ctr	Nef(+)-HIV-1	ΔNef-HIV-1	rNef/myr
mean	25.6	469.0	125.0	460.6	228.7	626.4
S.D.	15.0	64.4	14.4	26.5	31.9	11.6

Release of TNF-α by MDMs differentiated in HEMA culture condition w/o EPO and in M-CSF-differentiated MDMs treated with rNef/myr or infected *in vitro* with VSV-G pseudotyped HIV-1-expressing (Nef(+)-HIV-1) or not expressing the *nef* gene (ΔNef-HIV-1). The data are expressed as picograms/mL and the results are representative of three independent experiments.

Therefore we determined the dose/response of recombinant human TNF-α (rhTNF-α) on CD36 expression in M-CSF-differentiated MDMs. CD14-positive monocytes were cultivated for 5 days in the presence of M-CSF. TNF-α was added to the culture for the following three days at concentrations of 10, 3, 1 and 0.3 ng/mL. The results shown in Fig. 10C demonstrate a significant inhibition of CD36 expression induced by TNF-α although the lower concentration (0.3 ng/mL) does not produce a statistically significant effect.

Before to assess the role of TNF-α on Nef-induced inhibition of CD36 expression, we first evaluated the neutralizing capability of a polyclonal rabbit anti-human TNF-α antibody in a TNF-α-induced killing bioassay, by using the WEHI 164 cells [32]. The titration curve shown in Fig. 10D demonstrates that rhTNF-α, induced cell death down to a concentration of 0.019 ng/mL in presence of 1 μg/mL of the transcriptase blocker actinomycin D. The addition of 1 μg/mL of anti-human TNF-α antibody progressively reduced the TNF-α-induced cytotoxicity which is completely abolished at a concentration of 0.625 ng/mL (Fig. 10D). To verify the capability of the polyclonal rabbit anti-human TNF-α antibody to neutralize the CD36 downregulation by rhTNF-α on M-CSF-differentiated MDMs, 1 μg/mL of the antibody was added to the cell culture at the same time as the rhTNF-α and incubated for additional three days. The antibody was also added every 24 h before the flow cytometry analysis. Once again, results demonstrate the capability of TNF-α to significantly inhibit CD36 expression down to a concentration of 1 ng/mL, however this activity was abolished by the presence of anti-human TNF-α antibody (Fig. 10E).

**Figure 11:**
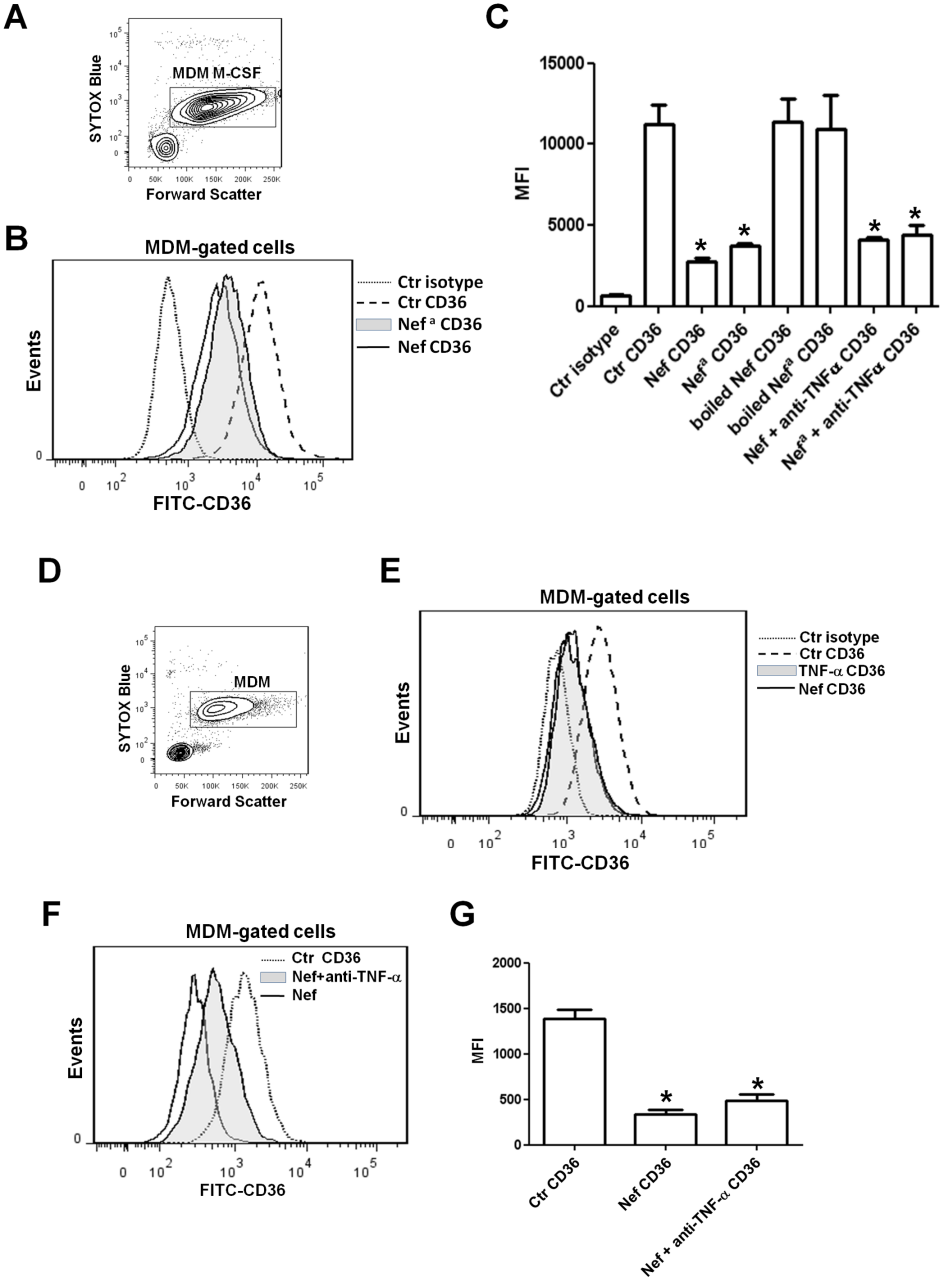
Nef-induced TNF-α release does not explain the downregulation of CD36 expression in MDMs. Cells isolated by using CD14 magnetic beads (Miltenyi Biotec) were cultured in presence of human M-CSF (10 ng/mL) for 5 days and for additional three days in presence of two rNef/myr from different manufactures, the inactivated rNef/myr proteins by boiling or in presence of the rNef/myr proteins together with anti-human TNF-α antibody (1 μg/mL). (A) The dot plot shows the viability of cells by using SYTOX Blue dead cell stain. MDMs are identified by rectangular gate (MDM M-CSF) and analyzed for CD36 expression by using FITC-conjugated CD36 antibody. (B) The relative fluorescence intensities of CD36 in Nef-treated (solid line), Nef^a^-treated (solid grey histogram) and untreated (dash line) cells are shown in the representative histogram. Matched isotype (dotted line) was used as control of non-specific fluorescence signals (Nef refers to the protein from Dr. M. Federico [22]; Nef^a^, to the protein from Jena Bioscience). (C) The column bar graph presents the MFI of untreated cells (Ctr CD36), Nef- and Nef^a^-treated cells (Nef CD36 and Nef^a^ CD36), cells incubated with inactivated Nef proteins (boiled Nef CD36 and boiled Nef^a^ CD36) and cells incubated with the Nef proteins and anti-humanTNF-α (Nef+anti TNFα CD36) stained with FITC-conjugated anti-CD36. The results (mean ± standard deviation) are representative of three independent experiments (*p<0.05). (D) PBMCs were cultivated in HEMA condition w/o EPO for three days and for additional three days in presence of TNF-α (10 ng/mL) or rNef/myr as control of CD36 downregulation. The viability of cells, assessed by SYTOX Blue dead cell stain, is shown in the panel D. MDMs are identified by rectangular gate (MDM) and analyzed for CD36 expression by using FITC-conjugated CD36 antibody. (E) The relative fluorescence intensities of CD36 in Nef-treated (solid line), TNF-α-treated (solid grey histogram) and untreated (dash line) cells are shown in the representative histogram. Matched isotype (dotted line) was used as control of non-specific fluorescence signals. The data shown are representative of three independent experiments with similar results. PBMCs were cultivated in HEMA condition without EPO for three days and for additional three days in presence of both polyclonal rabbit anti-human TNF-α (1 μg/mL) and rNef/myr or in presence of rNef/myr alone as control of CD36 downregulation. MDMs were then analyzed for CD36 expression by using FICT-conjugated CD36 antibody. (F) The histogram reports the relative fluorescence intensities of CD36 in Nef-treated (solid line), Nef- and anti TNF-α-treated (solid grey histogram) or untreated (dotted line) cells. SYTOX Blue was used to exclude dead cells. (G) The column bar graph represents the MFI of untreated cells (Ctr CD36), Nef-treated cells (Nef CD36), Nef- and anti-humanTNF-α-treated cells (Nef+anti TNF-α CD36) stained with FITC-conjugated CD36 antibody. The results (mean ± standard deviation) are representative of three independent experiments (*p<0.05).

To understand whether TNF-α released by MDMs treated with rNef/myr could have a role in CD36 downregulation, polyclonal rabbit anti human TNF-α antibody (1 μg/mL) was added to M-CSF-differentiated MDMs at the same time as rNef/myr and incubated for additional three days. The antibody was added every 24 h before the flow cytometry analysis. The Fig. 11 shows a representative dot plot (panel A) and histogram (panel B) of M-CSF-differentiated MDMs (dot plot) and MFI of CD36 expression levels in control cells and in cells treated with two rNef/myr from different source (histogram) as identified by “Nef”, obtained from the lab of Dr. M. Federico [22]; and “Nef^a^”, from Jena Bioscience. The level of CD36 inhibition is similar in cells treated with both the recombinant Nef proteins. In addition, as control for LPS contamination, the Nef proteins were inactivated by boiling and as shown in Fig. 11C. CD36 expression was not inhibited in cells treated with both the inactivated Nef proteins. Finally, the addition of anti-human TNF-α antibody was unable to significantly counteract the CD36 downregulation induced by Nef proteins (Fig. 11C). Similar experiments were performed in PBMCs cultivated in HEMA culture condition w/o EPO for three days (Fig. 11D). Recombinant human TNF-α (10 ng/mL) or rNef/myr (as experimental control) were added for additional 3 days and CD36 expression was analyzed by flow cytometry. According to previous reports CD36 expression is significantly inhibited by rhTNF-α and such inhibition is comparable to that observed in the presence of rNef/myr (Fig. 11E). To understand the role of Nef-induced release of TNF-α in CD36 downregulation, polyclonal rabbit anti-human TNF-α antibody (1 μg/mL) was added at the same time as rNef/myr to PBMCs cultivated in HEMA condition w/o EPO culture. The antibody was added again every 24 h before the flow cytometry analysis. In Fig. 11F is shown a representative histogram in which CD36 expression in the presence of rNef/myr and anti-human TNF-α results less inhibited than in cells treated with rNef/myr only. However, this partial effect of anti-humanTNF-α antibody did not result in statistically significant reduction of the Nef effect on CD36 (Fig. 11G).

Although some of the results here presented, and data reported in literature, suggest a possible role of TNF-α in mediating Nef activity, these last experiments would tend to exclude a possible relationship between Nef-induced TNF-α release and Nef-mediated CD36 downregulation.

## Discussion

In the present study we highlight the effect of rNef/myr on the expression of the CD36 membrane glycoprotein. We used the HEMA culture system to expand the analysis of CD36 expression in different cell populations: erythroblasts, lymphocytes, and MDMs [34]. In particular, we found a downregulation of CD36 expression in MDMs when rNef/myr was added to the culture. We also observed that this effect was highly specific, since other macrophage markers analyzed (CD14, CD11c, CD86, CD68, CD206, TLR2 and TLR4) were not downregulated. Furthermore, despite the erythroblasts express high level of CD36 receptor as the MDM population, Nef treatment did not elicit effects suggesting a cell specific response. Because of such discrepancy, we suppose that a reduced or absent uptake of the recombinant Nef by erythroblasts occurred, although we cannot rule out the existence of a more complex molecular mechanism that might involve a different cell physiology between erythroblasts and MDMs. However, it is important to point out that Nef protein concentration of 50 ng/mL used in all the experiments is slightly higher than those observed in the blood of HIV-infected patients and SIV-infected macaques [40], [43].

EPO, an essential component of HEMA culture, allows a massive expansion of erythroid population from PBMCs. However, we observed that removal of this factor from HEMA culture determined a significant reduced expansion of erythroblasts, favoring a relative increase of MDMs. Interesting, HEMA culture w/o EPO affects neither the phenotypic profile of MDMs nor, most important, the rNef/myr-dependent CD36 downregulation. This peculiarity allowed us to obtain a higher number of MDMs, which was helpful for carrying out better targeted analyses of the cells, in particular phagocytosis assays and RNA extraction from purified cells by FACS.

Previous reports have widely demonstrated that HIV-1 infection compromises the functionality of phagocytic cells favoring the reactivation and development of opportunistic infections during AIDS progression. The HIV-1 Nef protein, produced exclusively by Human and Simian Immunodeficiency Viruses, is considered a virus component playing a critical role in AIDS pathogenesis in HIV-infected humans. Nef influences cellular signaling pathways leading to the enhancement of viral replication, immune elusion and enhanced survival in T-cells and macrophages [12]. It also widely affects the innate immune system impairing oxidative burst response and phagocytosis in monocytes/macrophages from HIV-1 patients [14]–[16]. In this regard, studies on human alveolar macrophages from HIV-1 infected individuals demonstrate an impaired phagocytosis of *Pneumocystis Carinii* [44] that is also associated to a reduced oxidative burst response to the pathogen *in vitro* challenge [45]. Moreover, macrophages from HIV-1–infected patients show reduced apoptotic neutrophils phagocytosis [46], and infected MDMs are unable *in vitro* to engulf pathogens as *Candida albicans* and *Toxoplasma gondii,* [47] as well as FcγR and CR3 mediated phagocytosis of bacteria [48]. Mazzolini et al [49] also revealed that defect in phagocytosis in HIV-1–infected macrophages can be ascribed to a failure in focal delivery of intracellular membranes. The authors suggested that Nef protein is essential for phagocytosis inhibition, since it interacts with the AP1 complexes required for optimal phagosome formation. The overall picture that emerges from studies on the impairment of innate immune system by Nef is quite intricate. Nonetheless, the key role of Nef in this aspect of viral pathogenesis is evident. Here we report that Nef-induced CD36 downregulation in macrophage is associated to impaired scavenger activity with both significant decreased phagocytosis of fluorescent beads or GFP-producing *Salmonella typhimurium*, and reduced capability to internalize oxidized lipoproteins. In fact, the CD36 is a multifunctional surface receptor present on several mammalian cells and tissues. In particular it is also found on specialized phagocytes such as macrophages and on erythroid precursors [5]. Among its multiple cellular functions CD36 as scavenger receptor recognizes specific lipid and lipoprotein components of bacterial cell walls [50], and erythrocytes infected with *plasmodium falciparum* [51], [52]. These functional activities generate an immune response which leads to opsonin-independent pathogen internalization.

The mechanism by which Nef downregulates CD36 expression remains elusive. The time course required by Nef to inhibit CD36 membrane expression suggests an indirect effect, probably mediated by soluble factor(s) with autocrine/paracrine activity. These data are consistent with already described observations concerning the Nef-induced release of inflammatory factors from MDMs. A previous report describes experimental evidence supporting the hypothesis that IL-10 participates to the Nef-dependent inhibition of the superoxide anion (O_2_ **^−•^**) released by NADPH oxidase during the respiratory burst in U937 monoblastic cell line [15]. In addition, it has been shown that Nef induces secretion of chemotactic factors from primary human monocyte-macrophages, such as the CC-chemokines MIP-1α and MIP-1β [17] that correlates with the activation of AP-1, NF-κB, STAT1 and STAT3 transcription factors [19]–[22]. With regard to a possible relationship between Nef and CD36, recent studies have reported that TNF-α inhibits both CD36 membrane and mRNA expression through a reduction of PPARγ activation [42]. More recently Zamora et al. [39] have demonstrated that both TLR2 and TLR4 signals downregulate CD36 expression on peripheral blood monocytes and such inhibition is mediated by the TLR-induced cytokine TNF-α. They have also reported that LPS, Pam3CSK4 and FSL1 represent the TLR2 and TLR4 ligands able to induce CD36 downregulation. However, other factors have been described to decrease the expression of CD36. Indeed, TGF-β1 and TGF-β2 inhibit the expression of CD36 by inducing phosphorylation of p44 and p42 isoforms of MAP kinase. This leads to subsequent MAP kinase-mediated phosphorylation of PPARγ and, consequently, to decreased transcription of the PPARγ target gene CD36 [53]. In our study we found TNF-α release in the medium by cells treated with recombinant Nef or infected with VSV-G pseudotyped HIV-1-expressing Nef. We also observed that recombinant human TNF-α added to M-CSF-differentiated MDMs or MDMs obtained in HEMA culture condition was capable to inhibit CD36 expression. The data obtained in presence of polyclonal rabbit anti human TNF-α antibody suggest that Nef-induced TNF-α release only partially contributes to downregulation of CD36 expression in Nef-treated MDMs, although the results are not statistically significant. However, we do not definitively exclude the involvement of other intermediate factor(s) in Nef-induced CD36 downregulation and further investigation is warranted to confirm any hypothesis.

Several reports have provided evidence, both *in vitro* and in animal models, of the capacity of CD36 to bind and internalize OxLDL playing thus a role in atherosclerotic lesions formation [54]. Recent studies have reported that monocyte expression of CD36, whose transcription is primarily regulated by the nuclear receptor LXR, PPARγ and PXR (LXR: Liver × receptor; PPARγ: Peroxisome proliferator activated receptor γ; PXR: Pregnane × receptor), is markedly reduced by HIV infection. In fact, the transcription of CD36 gene is impaired in monocytes and the mRNA levels significantly correlate with those of PPARγ in HIV positive patients [55]. Interestingly the same authors demonstrated that HIV p17 hijacks a Rack-1/Jak-1/STAT-1 pathway in macrophages. In turn STAT-1 binds specific responsive elements on the promoter of nuclear receptors such as PPARγ determining increased levels of CD36 expression [56]. Hitherto several studies have analyzed the pathogenic effects of HIV-1 involving the modulation CD36 expression in monocyte/macrophage cells. However, discrepancies exist among many studies describing opposite effects of HIV-I on CD36 expression [55]–[59]. Two large cross-sectional studies by Feeney et al [60] and Meroni et al [61] are paradigmatic of these conflicting data in which decrease or increase of CD36 membrane expression on monocytes from HIV-positive patients compared to healthy donors are reported.

Here we describe that Nef-induced CD36 downregulation determines impairment of other scavenger activity such as reduced capability to internalize oxidized lipoproteins. This could imply repercussions for the pathogenesis of atherosclerosis and cardiovascular disease (CVD) in HIV patients [62]. Indeed, HIV infection and its pharmacological treatment are associated with dyslipidemia and increased risk of CVD. Several authors [63], [64] have observed higher levels of oxLDL in HIV-infected patients under ART. Furthermore, they have demonstrated an association between oxLDL and HIV-related lipodystrophy, suggesting that the reduction of LDL receptor levels might represent a possible cause. This hypothesis is substantiated by previous study demonstrating a lower LDL-receptor expression in lipodystrophic HIV-infected patients with respect to nonlipodystrophic HIV-infected patients [65]. Unfortunately, the *in vivo* implication and the role of Nef-mediated CD36 downregulation in determining or contributing to the onset of atherosclerosis and CVD are difficult to establish by the ART in HIV-infected patients. Indeed, several reports have demonstrated that ritonavir and other protease inhibitors (PIs) as part of ART alter the expression of CD36 [59], [65].

In conclusion HIV-1 infection compromises the functionality of phagocytic cells ultimately favoring the reactivation and development of opportunistic infections during AIDS progression. The data here presented reveal for the first time that soluble rNef/myr protein dramatically reduces CD36 surface expression on MDMs. Thereby, this new Nef activity could contribute to the strategies elaborated by HIV-1 to altered pathogen disease outcomes and support the onset of opportunistic infections in HIV-1 infected people. The molecular mechanisms underlying the effects of Nef-mediated CD36 downmodulation on AIDS pathogenesis are still to be fully clarified. Thus, a deeper knowledge of the mechanisms of Nef induced effects should be considered of primary importance for the development of intervention strategies and the advancement of new anti-HIV therapeutics. Unraveling factor(s) and mechanism(s) of action responsible for Nef effect might represent an exciting challenge in order to identify new pharmacological target(s) able to counteract severe opportunistic infections in HIV-1 patient amelioratating their pathologic conditions.
